# Cerebellar microcircuits enable robust evidence-based decisions through cortico–cerebellar coupling

**DOI:** 10.1073/pnas.2616911123

**Published:** 2026-08-27

**Authors:** Yeyao Bao, Liao Yu, Liangfu Lu, Zhuoqin Yang, Yunliang Zang

**Affiliations:** ^a^ Academy of Medical Engineering and Translational Medicine, Medical Faculty, Tianjin University, Tianjin 300072, China; ^b^ State Key Laboratory of Advanced Medical Materials and Medical Devices, Tianjin University, Tianjin 300072, China; ^c^ School of Mathematical Sciences, Beihang University, Beijing 100191, China

**Keywords:** cerebellar learning, decision-making, feedforward network, primacy bias, pattern separation

## Abstract

The cerebellum is increasingly implicated in perceptual decision-making, yet it remains unclear how the cerebellar cortex can support sparse evidence accumulation over behavioral time scales without assuming cortical-style dense local excitatory recurrence. We present a biologically constrained modeling framework showing that cerebellar microcircuits can implement graded accumulation and competition in the absence of such recurrence. In our model, type-II Purkinje-neuron excitability generates firing-rate hysteresis that prolongs the impact of brief inputs far beyond intrinsic membrane and synaptic time constants, enabling accumulation across long interevent intervals. Purkinje neuron collateral inhibition produces competitive divergence and tunes temporal evidence weighting, revealing a trade-off between commitment and primacy bias. In a bidirectionally coupled cortico-cerebello-cortical model, cerebellar processing reduces primacy while cortical processing reduces indecision, improving robustness. Finally, granule-layer sparsification improves the separability of correlated inputs, enhancing discrimination under biologically realistic stimulus statistics. Together, these simulation results propose a mechanistic division of labor that positions the cerebellum as an active computational partner in perceptual decisions.

## Significance

Traditionally associated with motor control, the cerebellum is increasingly recognized for its role in cognition. Yet how cerebellar cortical circuits can sustain evidence accumulation without assuming dense cortical-style local excitatory recurrence remains unclear. Using biologically constrained modeling, we show that Purkinje-neuron excitability and local inhibition enable graded accumulation and competition over behavioral timescales. When coupled with cortical circuits, cerebellar dynamics reduce primacy bias while cortical recurrence minimizes indecision, revealing a unified, distributed mechanism for robust decision-making.

The cerebellum has long served as a model system for linking cellular physiology, canonical microcircuit architecture, and computation. Classic theories emphasized sensorimotor control and learning, leveraging stereotyped connectivity and well-characterized neuronal dynamics (1–6). Converging evidence from anatomy, electrophysiology, and imaging now implicates the cerebellum in cognitive functions, including working memory and decision-making (7–15). A central challenge is to move from phenomenology to mechanism: what computations does cerebellar circuitry contribute outside the motor domain, and through which biophysical and microcircuit substrates?

Perceptual decision-making provides a stringent test because it requires integrating noisy, often sparse evidence over hundreds of milliseconds to seconds and converting that graded estimate into a categorical commitment (9, 10, 16–20). In cortical models, such long-timescale integration is typically attributed to strong local recurrent excitation (21–23), often supported by slow NMDA-mediated currents, and in other frameworks to multistage feedforward dynamics and distributed loops (21, 24–26). The cerebellar system also participates in long-range closed-loop interactions (27–30), but cerebellar cortical microcircuits are organized differently from canonical cortical integrators: synaptic time constants are fast and the cortex lacks dense, cortical-style local excitatory recurrence (1–3, 31–35). This raises a mechanistic puzzle in light of recent causal and correlational results. Rodent evidence-accumulation tasks recruit cerebellar pathways (7, 9, 10), Purkinje-neuron perturbations impair choice accuracy, and Purkinje neurons exhibit cue-period bidirectional modulation during accumulation (9, 10). Together, these observations suggest that cerebellar activity is not merely motoric or a passive readout of cortical decisions, but may participate in computations that shape choice.

Three questions follow. First, what cerebellum-specific neuronal and microcircuit mechanisms can expand the effective integration window to support sparse, stochastic evidence accumulation through mechanisms distinct from cortical-style dynamics? Second, if both cortex and cerebellum influence decisions, what computational advantage is gained by their interaction, and what division of labor makes the coupled system more robust than either module alone? Third, under biologically realistic input statistics—where evidence streams can be correlated (36–38)—can canonical granule-layer transformations improve separability and thereby promote robust competition and decision readout?

Here we develop a biologically constrained modeling framework grounded in rodent evidence-accumulation paradigms in which sensory evidence arrives as randomly timed pulses (9, 10, 19). We first analyze a cerebellar microcircuit model to identify intrinsic and local mechanisms sufficient for graded accumulation and competition. We then embed this circuit in coupled cortico-cerebellar architectures to ask how bidirectional interactions shape decision performance. By comparing cerebellar-only, cortico-cerebellar, cortico-cerebello-cortical, and cortex-only models, we isolate the roles of Purkinje-neuron excitability, Purkinje-neuron collateral inhibition, granule layer sparsification, and cortico-cerebellar coupling.

Within this framework, we identify an intrinsic Purkinje-neuron mechanism that extends the effect of brief inputs to support low-frequency accumulation, and a local inhibitory mechanism that tunes temporal evidence weighting, revealing a trade-off between commitment and primacy. We then show that closed-loop cortico–cerebellar coupling produces a division of labor: cerebellar dynamics mitigate primacy bias, whereas cortical recurrence reduces indecision, together improving robustness across stimulus statistics and timing variability. Finally, we relax the common assumption of independent evidence channels (21, 24, 39, 40) and consider correlated input streams (36–38); in this biologically realistic regime, granule-layer sparsification improves representational separability and enhances discrimination. We conclude by deriving testable predictions linking Purkinje-neuron collateral inhibition and Golgi-cell–mediated sparsification to measurable changes in temporal kernels, population representations, and choice behavior, and by predicting complementary cortical vs. cerebellar signatures across task regimes.

## Results

### Cerebellar Circuits Contribute to Evidence Accumulation in Decision-Making.

**Fig. 1.**
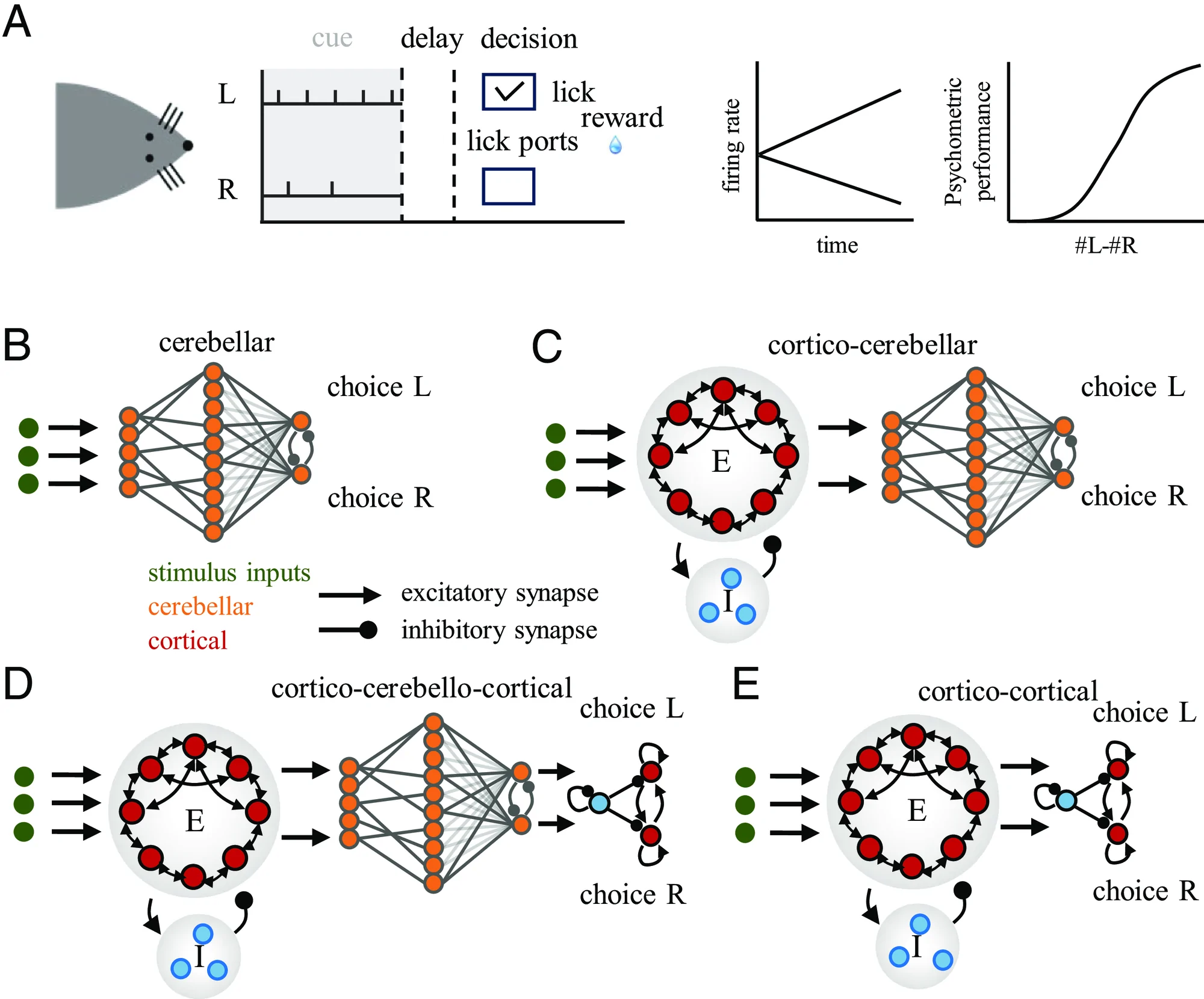
Experimental paradigms and alternative circuit models. (*A*) Rodent evidence-accumulation task. A fixed cue (evidence) period is followed by a delay and a choice report (lick to the side with more stimuli) (9, 10, 19). Schematics of Purkinje-neuron firing rates (*Middle*) and psychometric performance (*Right*) are shown. (*B*) Cerebellar-only model driven by sensory-triggered mossy-fiber spike bursts (41). Purkinje neurons are coupled by inhibitory axon collaterals (42–44). (*C*) Cortico-cerebellar model in which a cortical continuous-attractor module preprocesses sensory inputs before they enter the cerebellum (45). (*D*) Cortico-cerebello-cortical model extending (*C*) with a cortical decision module that integrates evidence and generates categorical choices (21). (*E*) Cortico-cortical model, matched to (*D*) but without the cerebellar module. The two cortical modules differ primarily in connectivity and parameter settings; schematics emphasize their distinct functions. See *Methods* Details.

To examine how cerebellar circuitry could support perceptual decisions, we modeled rodent evidence-accumulation paradigms that probe decisions under temporal uncertainty (Fig. 1*A*). We focus on the fixed-time task, in which stochastic air puffs are delivered to left and right whiskers during a fixed cue epoch, followed by a delay and a choice report. Trained animals achieve high accuracy, and Purkinje neurons exhibit cue-period ramping or bidirectional modulation of firing rates (9, 10). We also simulated the reaction-time task (Fig. 6 and *SI Appendix*, Fig. S6), in which cue duration is variable and animals respond as soon as they infer which side has the higher Poisson input rate (36).

Across models, we do not explicitly simulate motor execution. Instead, a “decision” is registered when the firing-rate difference between two competing populations exceeds a fixed threshold (46), allowing a common readout across architectures.

We compared four circuit architectures (Fig. 1 *B*–*E*). The pure cerebellar model isolates intrinsic and local circuit mechanisms for accumulation and competition, with mossy fibers providing discrete spiking bursts motivated by bouton recordings (41). The cortico–cerebellar model adds a cortical continuous-attractor module that generates heterogeneous, temporally dispersed mossy-fiber activity consistent with imaging data (45, 47, 48). The cortico-cerebello-cortical model closes the loop by adding a cortical decision network (21) downstream of the cerebellum (49–51), enabling us to test how coupling two partially redundant decision-capable modules changes performance. A cortex-only (cortico–cortical) model serves as a reference for quantifying the computational advantages conferred by cerebellar involvement compared to cortical-only processing.

Together, these models provide a controlled framework to ask i) how cerebellar microcircuits can integrate sparse evidence despite fast synapses and short membrane time constants, and ii) what computational advantage emerges from cortico-cerebellar coupling.

### Type-II Excitability Enables Low-Frequency Accumulation in Purkinje Neurons.

Neuronal excitability strongly shapes input–output transformations, yet it is often abstracted away in circuit and cognitive models. Purkinje neurons are the sole output of the cerebellar cortex and exhibit complex intrinsic dynamics. Experimental recordings indicate that Purkinje neurons typically exhibit type-II excitability, characterized by a finite onset firing rate (nonzero minimum firing frequency) and a discontinuity in the frequency–current (f–I) relationship under sustained current injection, in contrast to the continuous f–I relationship observed in type-I excitability (52–54). Here, the type-I model serves as a matched computational control to isolate the contribution of type-II dynamics, rather than to represent a distinct biological Purkinje-cell class. Both models use the same membrane-potential equation (*Methods*) but different parameter regimes that determine excitability class (*SI Appendix*, Fig. S1).

**Fig. 2.**
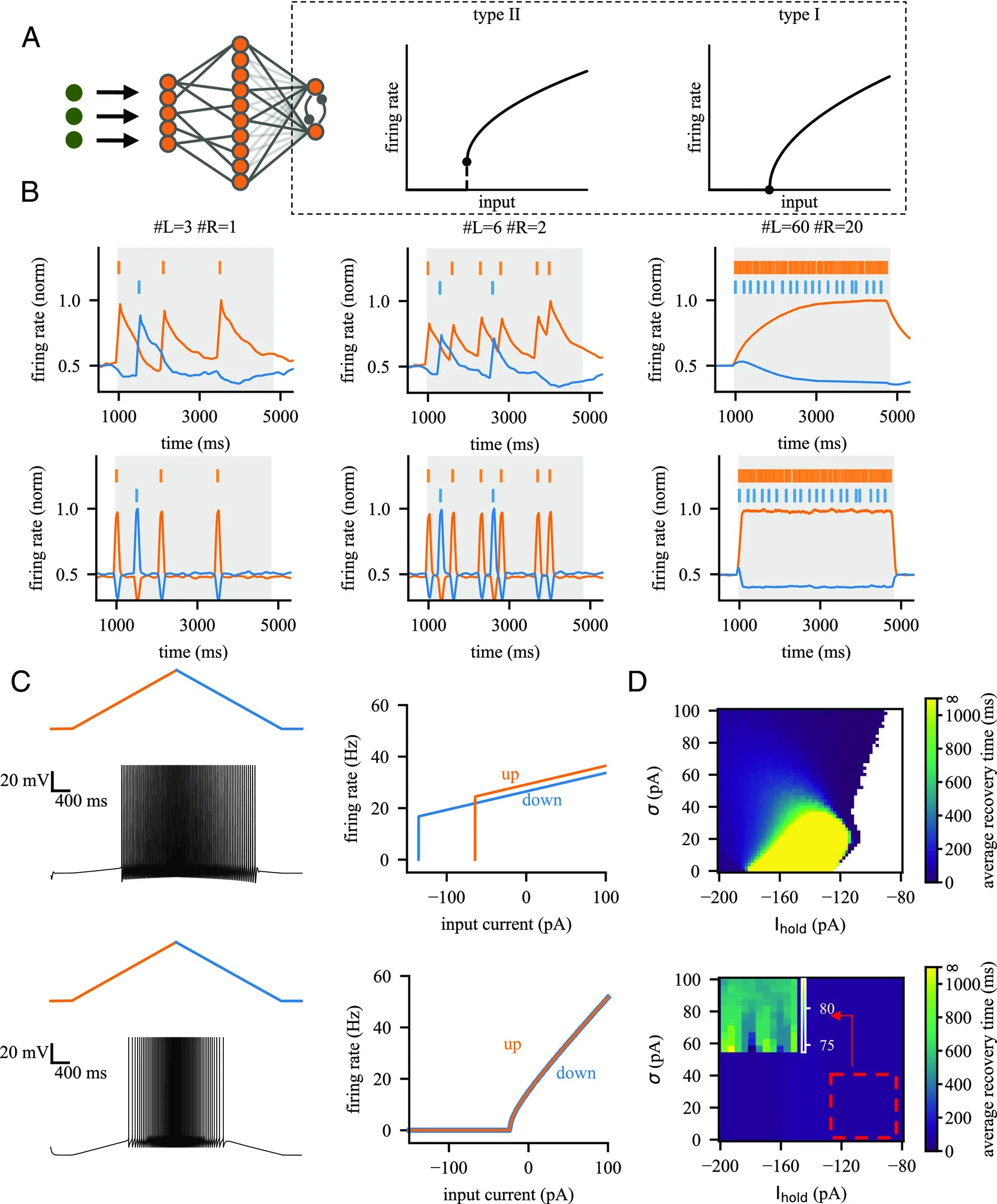
Firing rate hysteresis extends the integration window and enables low-frequency evidence integration. (*A*) Cerebellar circuit model in which Purkinje neurons operate in either a type-II excitability regime or a type-I regime used as a matched computational control. The type-I and type-II regimes exhibit continuous vs. discontinuous frequency–current (f–I) relationships, respectively. (*B*) Purkinje-neuron firing-rate dynamics under increasing event rates (*Left* to *Right*). Vertical lines mark stimulus events; traces show firing rates of *Left*- and *Right*-selective populations. *Top*: type-II regime. *Bottom*: type-I regime. (*C*) Firing-rate hysteresis in type-II regime during slow current ramps (*Top*), contrasted with the history-independent, continuous response in the type-I regime (*Bottom*). (*D*) Recovery time constant as a function of $I_{\mathrm{hold}}$ and noise variance σ. The white region indicates regimes where neurons are entirely in the firing regime at baseline and never return to silence.

We first asked whether the cerebellar circuit model with type-II Purkinje neurons can integrate sparse sensory events in the absence of cortical-like mechanisms. In the pure cerebellar model, left- and right-selective mossy-fiber populations deliver brief spike bursts representing discrete sensory events (41) (Fig. 2). Granule cell→Purkinje neuron synapses were modeled as AMPA conductances with a 2 ms decay time constant, and Purkinje neuron model had an intrinsic integration time constant of ∼32 ms (55–57). Inhibitory axonal collaterals between Purkinje neurons were modeled as GABA conductances (42, 43).

In the type-II model, consistent with experiments (9, 10), population activity ramps during the cue period: the population receiving stronger evidence increases firing while the competing population decreases firing. Notably, ramping persists even when events are extremely sparse (0.8 Hz; interevent interval 1,250 ms), far exceeding intrinsic membrane and synaptic time constants.

In contrast, replacing type-II regime with the type-I regime abolishes low-frequency accumulation. Type-I models only show effective accumulation at high event rates when interevent intervals are shorter than intrinsic time constants; at experimentally tested rates [2.5 to 5.0 Hz (9, 10, 19)], stimulus-evoked responses completely recover before the next event, and activity fails to persist after cue period required by the fixed-time task for decision categorization (9, 10, 19).

Purkinje neuron collateral inhibition generates opposing population trajectories during accumulation; removing this inhibition eliminates competitive divergence (*SI Appendix*, Fig. S2). These results link sparse-event accumulation to type-II excitability and motivate a mechanistic question: which feature of type-II dynamics extends the effective integration window?

### Firing-Rate Hysteresis Extends the Temporal Window of Neuronal Integration.

To identify the mechanism, we analyzed the near-threshold input–output dynamics of the type-I and type-II models. Type-II models exhibit firing-rate hysteresis during slow current ramps (Fig. 2*C*), consistent with experimentally observed distinct “up” and “down” firing regimes (52–54). In contrast, type-I models show a single-valued, history-independent firing-rate curve.

We quantified the functional consequence of hysteresis by applying a holding current ($I_{\mathrm{hold}}$) and Gaussian noise (variance σ), and then measuring recovery time following a single transient stimulus (Fig. 2*D*). In the type-II model, once a stimulus drives a neuron into the firing regime, returning to silence requires a larger opposing drive, producing slow recovery at the population level. Recovery time constants frequently exceed 1 s across a broad parameter range, orders of magnitude longer than the tens-of-milliseconds recovery observed in the type-I model.

Type-I models could not reproduce this slow recovery because their upward and downward thresholds are symmetric (*SI Appendix*, Fig. S3). Thus, the firing-rate hysteresis, a well-defined experimental feature of Purkinje neurons (52–54), provides a candidate single-cell mechanism that prolongs the impact of brief inputs and supports sparse-event accumulation.

### Cortical Preprocessing Enhances Cerebellar Integration.

**Fig. 3.**
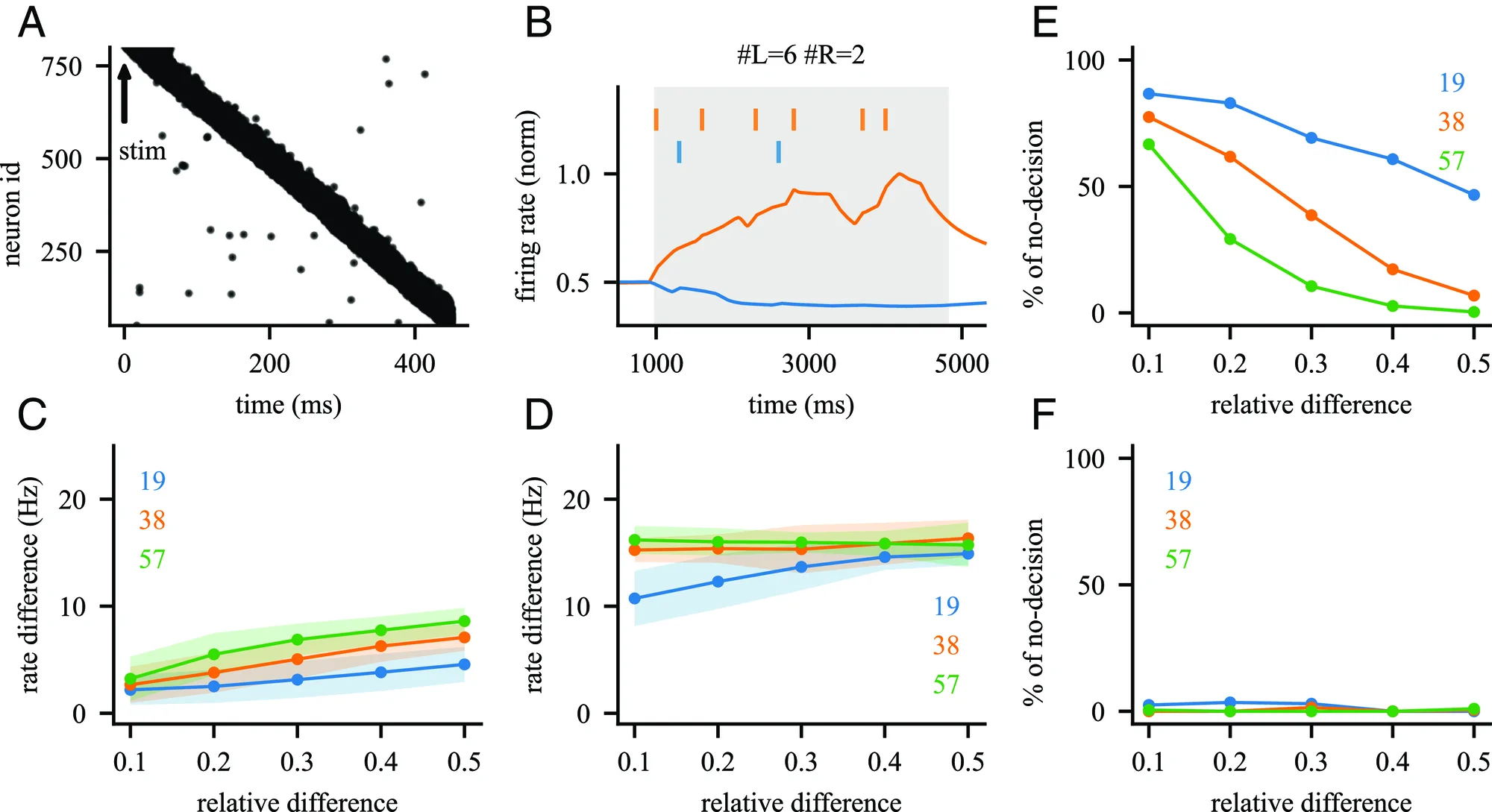
Cortical preprocessing enhances cerebellar evidence accumulation. (*A*) Example mossy-fiber spike trains with heterogeneous latencies generated by the cortical continuous attractor module. (*B*) Purkinje-neuron ramping under low-frequency evidence. (*C* and *D*) Decision readiness (cue-end firing-rate difference) for spike bursts (*C*) versus cortical-preprocessed inputs (*D*). (*E* and *F*) Proportion of undecided trials as a function of relative evidence difference for direct bursts (*E*) versus preprocessed inputs (*F*). Colored numbers indicate total evidence across both sides.

In the previous cerebellar model, each sensory event is represented as a brief mossy-fiber burst (41). However, a single sensory event can elicit heterogeneous and delayed population responses upstream, effectively distributing evidence over time (45, 47, 48). To examine how such preprocessing affects cerebellar accumulation, we added a simplified cortical module with continuous-attractor dynamics (58, 59). In this context, an attractor is a stable activity pattern sustained by recurrent circuitry; a continuous attractor supports a family of nearby stable states, enabling graded, persistent population responses rather than an all-or-none transition. We use this module as a minimal, generic mechanism for transforming punctate inputs into temporally extended and heterogeneous drive (58, 59): recurrent excitation sustains activity, while spike-frequency adaptation and inhibition shape its temporal dispersion (Fig. 3*A*).

When these preprocessed inputs drive the cerebellar circuit, Purkinje-neuron ramping becomes more pronounced under low event rates (compare Fig. 3*B* with Fig. 2*B*). Cortical preprocessing increases the firing-rate separation between competing Purkinje neuron populations at cue end (Fig. 3 *C* and *D*), thereby increasing “decision readiness” under a fixed-threshold readout.

Consistent with increased cue-end separation, cortical preprocessing reduces the fraction of undecided trials, particularly at low event rates (Fig. 3 *E* and *F*). These simulation results suggest a division of labor: cortical preprocessing distributes evidence over time, while cerebellar hysteresis integrates and stabilizes it.

### Purkinje-Neuron Mutual Inhibition Tunes Temporal Evidence Weighting.

**Fig. 4.**
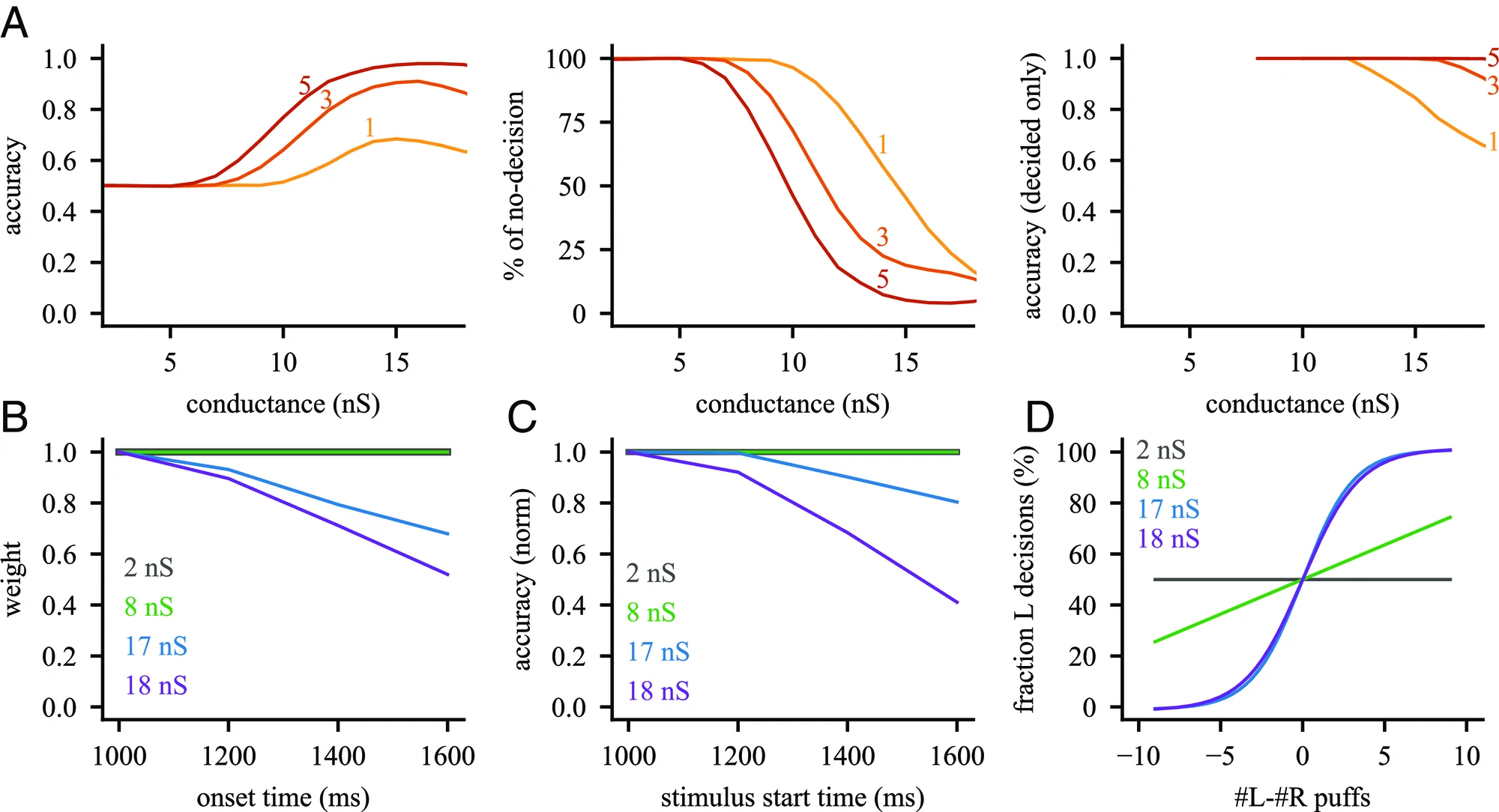
Mutual inhibition shapes accumulation and temporal weighting. (*A*) Psychometric curves versus mutual inhibition conductance between Purkinje neurons. *Left*: accuracy including undecided trials. *Middle*: undecided fraction. *Right*: accuracy excluding undecided trials. Colors indicate evidence-count differences. (*B*) Temporal weighting (psychophysical kernel) of a pulse as a function of pulse time. (*C*) Accuracy versus preferred-stream onset time (nonpreferred onset fixed). (*D*) Psychometric curves versus relative evidence difference. Colors in *A* indicate the evidence difference between the two sides. Colors in *B*–*D* indicate inhibition strength.

We next examined how local mutual inhibition mediated by Purkinje neuron axon collaterals shapes competition and temporal weighting. When inhibitory conductance exceeds ∼18 nS, network activity becomes unstable and enters self-sustained bistable states even without sensory drive (*SI Appendix*, Fig. S4) (60). Within the stable regime, psychometric performance varies nonmonotonically with inhibition strength (Fig. 4*A*).

Weak inhibition increases trial-to-trial variability in cue-end separation and increases the fraction of undecided trials, reducing overall accuracy (Fig. 4 *A*, *Left* and *Middle*). Conditioning on committed trials reveals that weak inhibition could improve accuracy among decided trials (Fig. 4 *A*, *Right*), suggesting a trade-off between commitment rate and integration quality.

To examine how inhibition strength regulates integration quality, we assessed temporal weighting using a psychophysical pulse paradigm (61): transient pulses delivered at variable latencies on top of a constant background reveal a primacy-like weighting profile (Fig. 4*B*) (62). Weak inhibition attenuates primacy, producing more uniform weighting across time. This reduced temporal bias improves robustness to stochastic evidence timing: when the nonpreferred stream begins at a fixed time and the preferred stream is delayed, strong inhibition causes pronounced accuracy loss whereas weak inhibition preserves performance (Fig. 4*C*). However, weak inhibition yields shallower psychometric curves because more trials fail to reach the decision threshold (Fig. 4*D*).

These simulation results suggest that local mutual inhibition among Purkinje neurons can act as a control parameter that trades off commitment (fewer undecided trials) against temporal bias (stronger primacy).

### Cortico-Cerebello-Cortical Coupling Balances Primacy Bias and Indecision.

Motivated by higher-order cerebello-cortical feedback pathways (7, 63–66), we next asked whether adding a downstream cortical decision module can resolve the bias–indecision trade-off. We therefore coupled the cerebellar circuit to a cortical decision module (21), yielding a cortico-cerebello-cortical model, and also compared it to a cortex-only model with matched cortical dynamics.

**Fig. 5.**
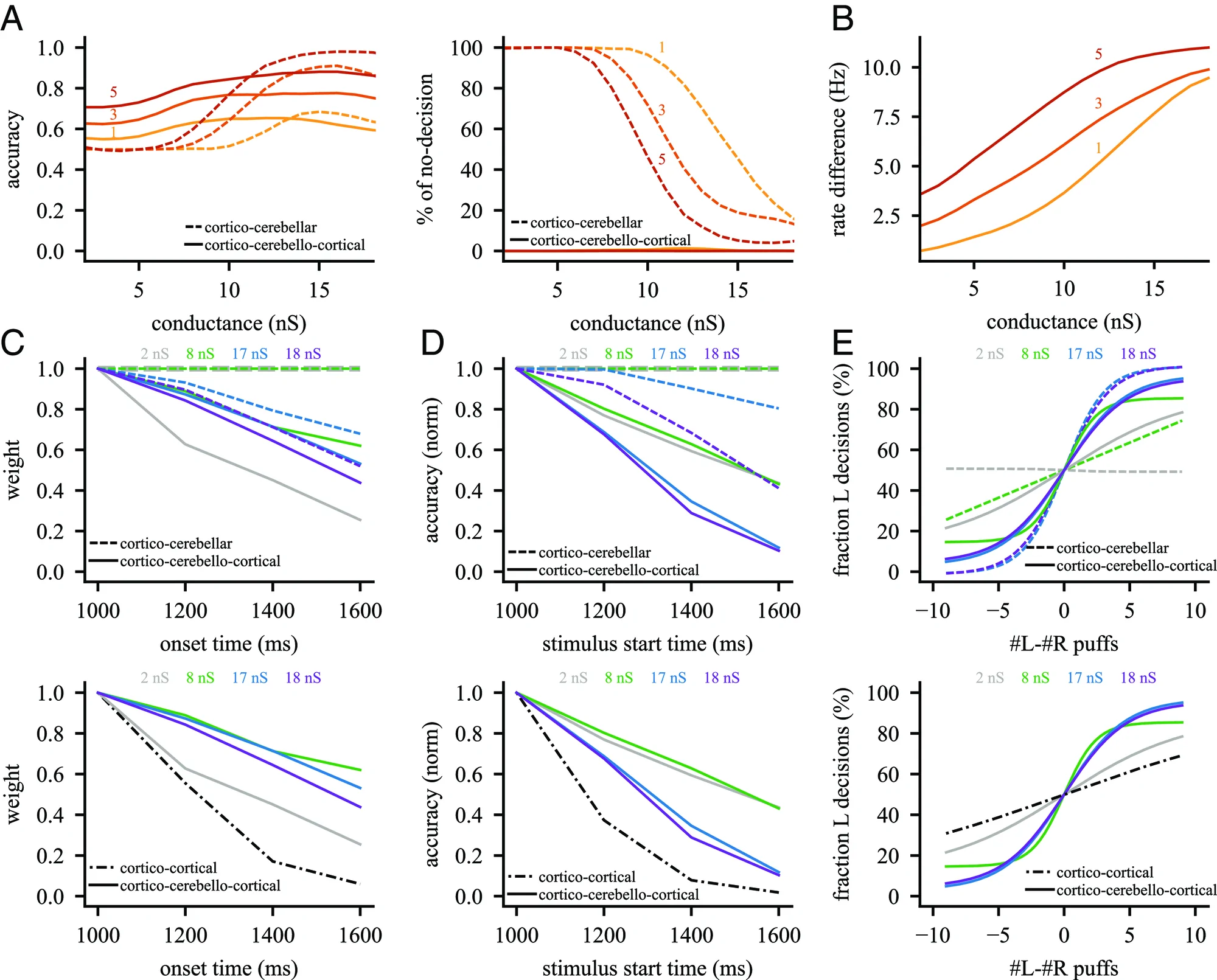
Closed-loop coupling balances temporal bias and commitment. (*A*) Psychometric curves vs. Purkinje neuron mutual inhibition conductance. *Left*: accuracy including undecided trials. *Right*: undecided fraction. (*B*) Cue-end decision readiness vs. mutual inhibition conductance. (*C*) Temporal weighting vs. pulse time. (*D*) Accuracy vs. preferred-stream onset time (nonpreferred fixed). (*E*) Psychometric curves vs. relative evidence difference. In *A* and *B*, colors indicate evidence-count differences. In *C*–*E*, colors indicate Purkinje neuron inhibition strength; solid: cortico-cerebello-cortical; dashed: cortico-cerebellar; dash-dotted: cortex-only. *Top*: comparison of the cortico–cerebello–cortical model with the cortico–cerebellar model. *Bottom*: comparison of the cortico–cerebello–cortical model with the cortico–cortical model.

Adding the cortical module reduces undecided trials and improves accuracy in the weak-inhibition regime relative to the cortico-cerebellar model, although performance decreases slightly at high Purkinje neuron inhibition range (Fig. 5*A*). Across inhibition values, stronger Purkinje neuron inhibition increases cue-end separation (Fig. 5*B*) but also increases primacy bias (Fig. 5 *C* and *D*). Notably, when mutual inhibition is weak (2 nS) in the cortico–cerebello–cortical model, the cerebellar module primarily relays input and contributes little accumulation; accordingly, primacy in this regime is dominated by the downstream cortical dynamics.

Across the stable conductance range, temporal bias ranks consistently: the cortico-cerebellar model shows the weakest primacy, the cortico-cerebello-cortical model is intermediate, and the cortex-only model shows the strongest primacy (Fig. 5 *C* and *D*). Consistent with this complementarity, the coupled model achieves high performance across a broader range of Purkinje neuron inhibition strengths than either module alone (Fig. 5*E*).

These simulation results support a computational division of labor: cerebellar dynamics reduce temporal bias during evidence integration, whereas the cortical module promotes categorical commitment and reduces indecision. Example circuit dynamics illustrating these roles are shown in *SI Appendix*, Fig. S5.

### Cortico-Cerebello-Cortical Model Reproduces Behavioral and Neuronal Signatures.

**Fig. 6.**
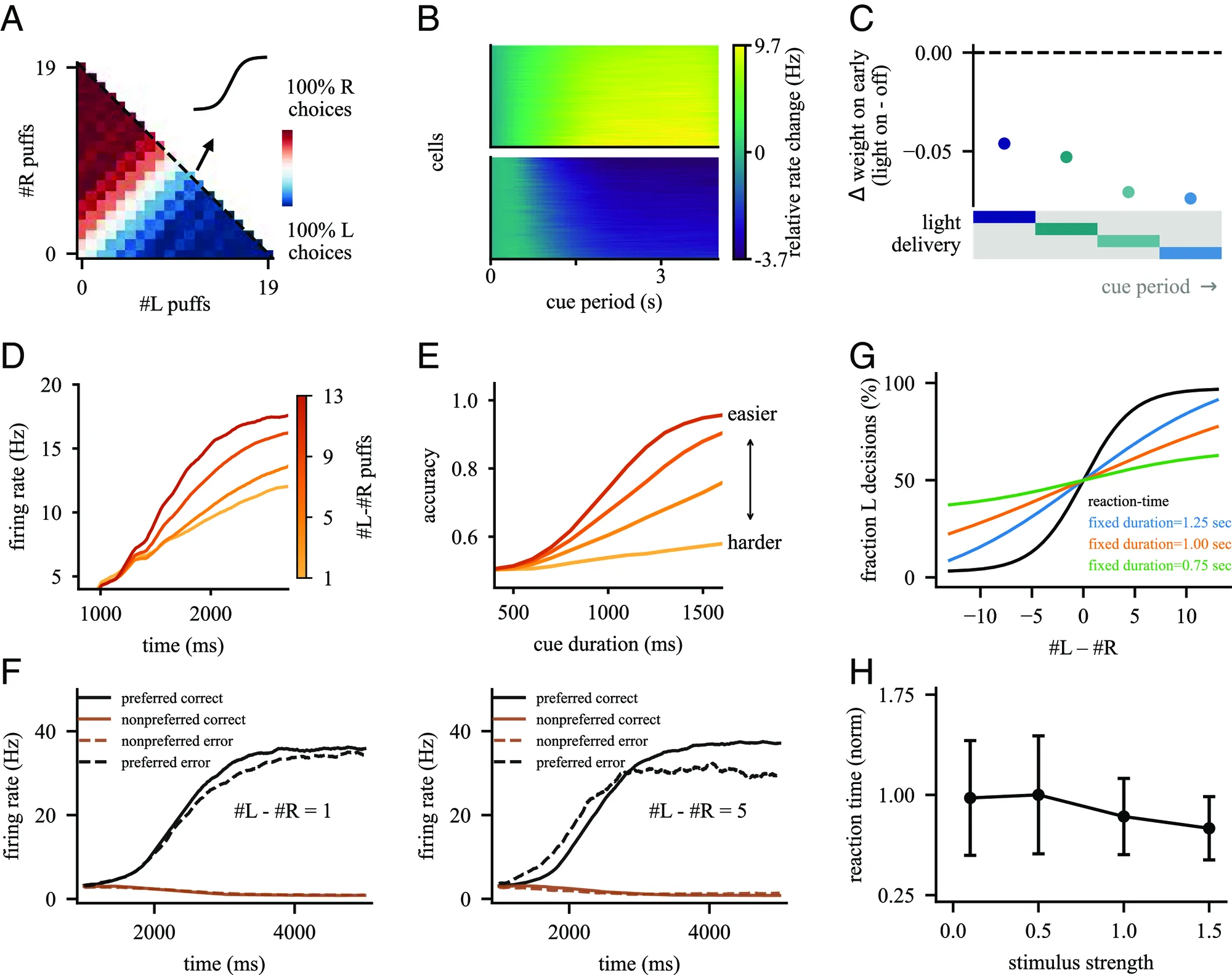
Evidence-accumulation dynamics captured by the cortico-cerebello-cortical model. (*A*) Choice probability as a function of *Left* and *Right* puff counts. (*B*) Cue-period activity of winning (*Top*) and losing (*Bottom*) Purkinje neuron populations. (*C*) Weight change following cue-period Purkinje neuron perturbations applied at different times. (*D*) Winning-population Purkinje neuron trajectories across evidence differences. (*E*) Psychometric curves across cue durations and difficulty levels. (*F*) Population trajectories for correct and error trials across stimulus differences. (*G*) Psychometric curves comparing different fixed cue durations with the reaction-time paradigm. (*H*) Mean reaction time changes vs. stimulus strength. Panels *A*–*F*: fixed-time; panel *H*: reaction-time; panel *G*: both.

We next asked whether the cortico-cerebello-cortical model reproduces experimentally established hallmark behavioral and neural signatures of evidence accumulation. In the fixed-time task, the model reproduces choice probabilities as a function of left and right puff counts (Fig. 6*A*) (9). Purkinje neuron activity ramps during the cue period, with the winning population increasing and the losing population decreasing (Fig. 6*B*) (9). Cue-period perturbations of Purkinje neuron activity reduces accuracy, with later perturbations producing larger impairments, consistent with experimentally observed evidence discounting (Fig. 6*C*) (9). The slope of the winning trajectory scales with evidence strength (Fig. 6*D*) (9), and accuracy increases with cue duration for difficult discriminations while saturating for easier ones (Fig. 6*E*) (19). Population trajectories capture correct vs. error dynamics: at small evidence differences, trajectories are similar across trial types, whereas larger differences produce stronger divergence with reduced slopes on error trials (Fig. 6*F*) (65, 67).

In the reaction-time task, decisions are registered once the firing-rate difference crosses a threshold (36, 46). The model reproduces improved performance relative to short fixed cue durations (Fig. 6*G*) (19, 65), and mean reaction time decreases with increasing stimulus strength (Fig. 6*H*) (36). Notably, all these experimental signatures were already reproduced by the cortico-cerebellar model (*SI Appendix*, Fig. S6).

### Granule-Layer Sparsification Improves Decision Robustness Under Overlapping Input Representations.

The analyses above focused on cerebellar contributions to temporal integration and weighting. We next turn to the spatial dimension, which becomes critical under biologically realistic input statistics in which evidence streams are not perfectly separable. Such regimes can arise when sensory features share common drive or are encoded by partially overlapping neural populations, leading to correlated inputs and reducing the separability required for reliable competition and readout (36–38). This section therefore addresses our third question: whether canonical granule-layer transformations can improve separability and thereby promote robust decisions.

**Fig. 7.**
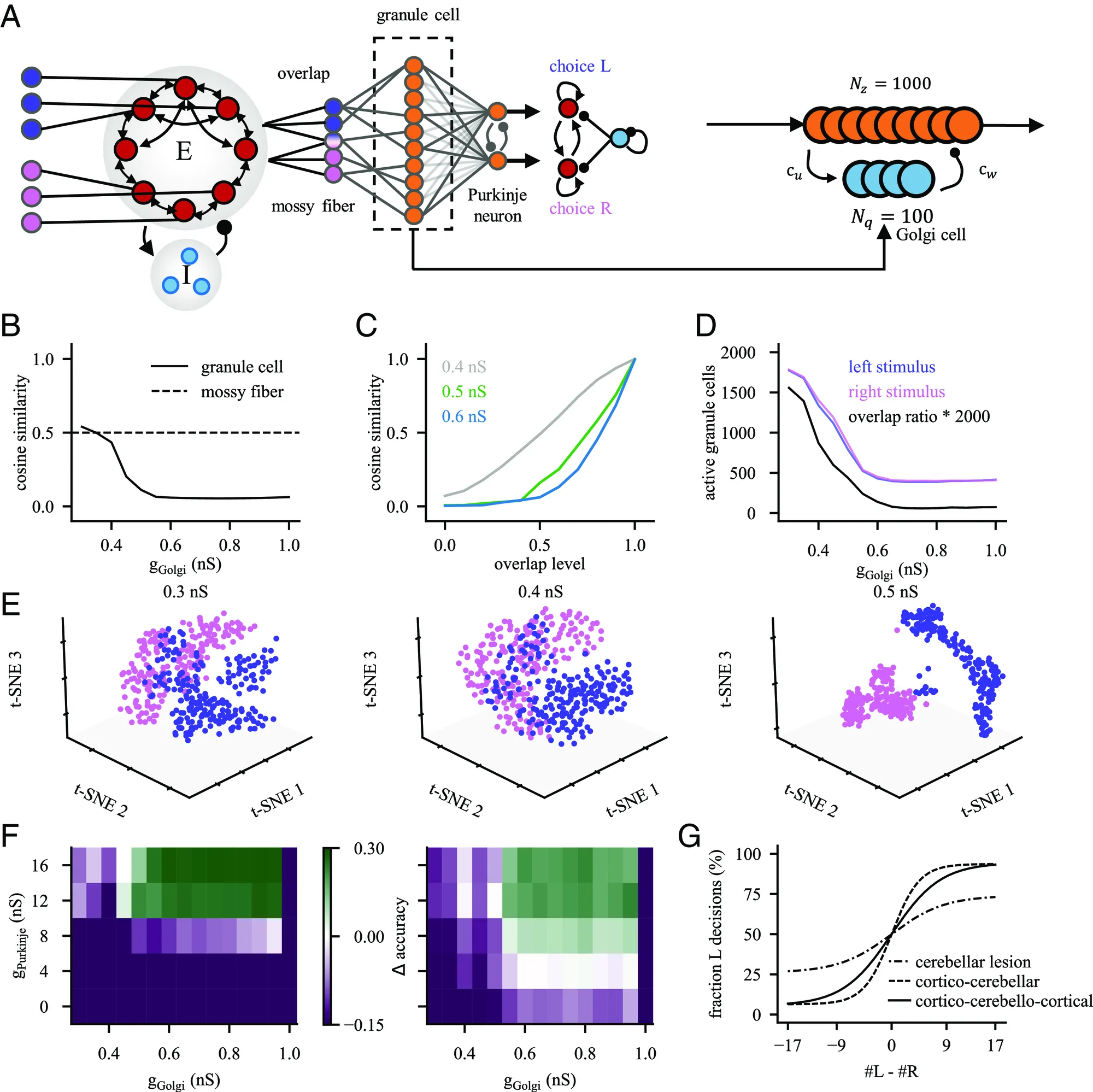
Cerebellar pattern separation improves decision reliability under overlapping inputs. (*A*) Mixed mossy-fiber encoding of *Left* and *Right* evidence; granule-cell sparsity is controlled by Golgi cell feedback inhibition. (*B*) Granule-cell response similarity vs. Golgi cell conductance (dashed: mossy-fiber input similarity). (*C*) Granule-cell response similarity versus the fraction of mixed mossy fibers; colors indicate Golgi-cell conductance. (*D*) Number of active granule cells and response overlap versus Golgi-cell conductance. (*E*) t-SNE visualization of granule-cell population responses as Golgi cell inhibition increases. (*F*) Accuracy gains relative to the cortex-only model across Golgi-cell and Purkinje-neuron inhibition strengths (*Left*: cortico-cerebellar minus cortex-only; *Right*: cortico-cerebello-cortical minus cortex-only). (*G*) Psychometric performance vs. stimulus strength across models. “Cerebellar lesion” corresponds to cortico-cortical model; The parameters corresponding to the other curves are $g_{\mathrm{Golgi}}=0.8\;\mathrm{nS},\;g_{\mathrm{Purkinje}}=12\;\mathrm{nS}$.

Building on established granule-layer pattern-separation principles (32, 34), we introduced overlapping representations at the mossy-fiber level by assigning subsets of mossy fibers to respond preferentially to left, right, or both evidence streams (Fig. 7*A*), thereby inducing correlated input patterns. In the cerebellar cortex, granule cells receive sparse mossy-fiber inputs and are regulated by Golgi-cell inhibition. Increasing Golgi cell inhibition produces sparser granule cell activation, reduces response similarity relative to mossy-fiber inputs, and enhances pattern separation (Fig. 7 *B*–*D*). Consistent with this, t-SNE embeddings of granule cell population responses show progressively improved separation of left vs. right patterns with stronger Golgi cell inhibition (Fig. 7*E*). Improved separation increases discrimination and accumulation performance when mossy-fiber inputs are overlapped (Fig. 7 *F* and *G*).

Across a broad range of Golgi cell and Purkinje neuron inhibition strengths, the cortico-cerebellar model outperforms the cortex-only model. The full cortico-cerebello-cortical model maintains robust performance across a wider parameter range, though with smaller peak gains. These conclusions hold across overlap ratios (*SI Appendix*, Fig. S7). By contrast, the cortex-only model does not reach ceiling accuracy even at high evidence differences, consistent with experimental observations (9).

## Discussion

This study proposes a mechanistic account of how cerebellar microcircuits can contribute directly to perceptual decision-making. Our simulations identify two complementary computational dimensions that promote robust evidence-based decisions. Along the temporal dimension, intrinsic Purkinje-neuron dynamics can provide a slow, history-dependent state variable that supports sparse-event accumulation despite fast synapses and short membrane time constants. Local inhibition among Purkinje neurons drives competitive divergence and tunes the temporal weighting of evidence. Bidirectional cortico-cerebello-cortical coupling can resolve a trade-off between temporal bias and categorical commitment, improving robustness across stimulus statistics and temporal uncertainty. Along the spatial dimension, granule-layer sparsification decorrelates sensory inputs and increases the separability of choice-relevant representations, thereby enhancing discrimination when evidence streams are correlated.

A central challenge for accumulation under naturalistic conditions is that evidence often arrives as sparse, stochastic events whose interevent intervals exceed intrinsic neuronal and synaptic integration time constants—typically tens of milliseconds (55–57). Distinct from cortical strategies (21, 24, 26, 68), cerebellar microcircuits do not prominently feature strong local recurrent excitation, motivating the question of how they could support sparse evidence integration. Our simulations identify firing-rate hysteresis associated with type-II Purkinje-neuron excitability (52–54) as a candidate solution: brief inputs can trigger recovery times on the order of seconds, effectively extending the temporal footprint of each event and enabling accumulation across long interevent intervals. More broadly, this suggests a possible neuronal strategy for expanding integration windows that is distinct from cortical-style reverberation and may generalize to other circuits whose principal neurons exhibit type-II excitability (69, 70).

Beyond intrinsic dynamics, our model assigns a functional role to Purkinje-neuron axon collaterals. While these connections have been studied in the context of synchrony and development (42–44, 71), their computational contribution remains unclear. In our framework, local mutual inhibition among Purkinje neurons generates competitive divergence between populations representing alternative evidence streams and tunes temporal weighting. Increasing collateral inhibition strengthens cue-end separation and reduces indecision but also increases primacy bias and can destabilize dynamics at high conductance (*SI Appendix*, Fig. S4). We therefore interpret Purkinje neuron collateral inhibition as a microcircuit mechanism that shapes the psychophysical kernel, trading off commitment against temporal bias.

The cerebellum also operates within distributed loops. Mossy-fiber inputs reflect transformations performed by upstream forebrain circuits, and imaging suggests that pontine inputs can be temporally dispersed and heterogeneous (45, 47, 48). In our model, a cortical preprocessing module distributes each sensory event over time, increasing cue-end separation and reducing undecided trials, particularly under sparse evidence; this facilitation persists in the closed-loop cortico-cerebello-cortical model (*SI Appendix*, Fig. S8). Together, these simulations support a complementary interaction in which cortical dynamics diversify and temporally extend evidence representations, while cerebellar hysteresis integrates and stabilizes them. Importantly, preprocessing does not replace hysteretic Purkinje neuron dynamics: the type-II regime remains required for low-frequency accumulation (*SI Appendix*, Fig. S9).

Recent work has proposed that sequential, rather than persistent, activity can encode accumulated evidence through spatiotemporally tiled population dynamics (72). Our framework is compatible with this broader idea in that temporally structured input patterns—whether generated by a continuous attractor as modeled here or by alternative sequential-coding mechanisms—can be converted into stable, graded decision variables by cerebellar microcircuit dynamics. An important next step will be to test how different upstream coding schemes for evidence (persistent, sequential, or mixed) interact with cerebellar integration mechanisms to shape psychophysical kernels and neural trajectories.

At the systems level, we find that bidirectional cortico-cerebellar coupling can improve robustness by combining complementary failure modes. In the coupled cortico-cerebello-cortical model, cerebellar dynamics reduce primacy bias relative to cortex-only integration, whereas the cortical module reduces indecision near the choice boundary. This division of labor yields strong performance across a broader parameter range than either module alone, consistent with the idea that distributed circuits can implement similar computations through distinct mechanisms and thereby increase robustness to changes in input statistics and circuit state.

Finally, our theoretical results highlight a cerebellar advantage when evidence representations are overlapping or correlated. Many decision models assume distinct populations for each alternative (21, 24, 39, 40), but real sensory pathways often carry mixed signals: mossy fibers and other precerebellar nuclei can receive bilateral and convergent inputs, and in modalities such as audition, left/right evidence cannot be perfectly segregated (36–38). In this regime, correlated inputs can degrade discrimination in cortex-only models. In contrast, Golgi cell-controlled sparsification in the granule layer decorrelates mixed mossy-fiber patterns and improves separability, extending classical expansion-recoding ideas (73, 74) to decision computations. This suggests a general role for cerebellar pattern separation in cognitive settings where evidence streams are ambiguous or not cleanly partitioned.

### Limitations and Future Directions.

Recent experiments indicate that perceptual decision-making engages distributed, brain-wide activity patterns (7), although the precise computational roles of many regions remain unresolved. Our models intentionally abstract away cerebellum-related long-range loops (27–30), explicit thalamic relay dynamics (75–77), and contributions from basal ganglia and other regions (7, 78), and we use a threshold-based readout rather than an explicit motor implementation (46, 79). These simplifications allow us to isolate cerebellar microcircuit mechanisms, but an important next step is to embed the present framework within more complete architectures and test whether the proposed division of labor persists under richer motor and state-dependent constraints.

Core predictions can be tested experimentally: manipulating Purkinje neuron axon-collateral inhibition should bidirectionally modulate primacy in the psychophysical kernel and commitment rate; Golgi-cell perturbations (80) should preferentially impair performance when left/right evidence streams are correlated; and simultaneous cerebellar–cortical recordings during decision-making tasks should reveal cerebellar signatures associated with reduced temporal bias alongside cortical dynamics associated with categorical commitment.

## Methods

### Cortical Continuous Attractor Module.

The model was built on the attractor-based decision-making framework (21), with the continuous-attractor architecture, spike-frequency adaptation, and connectivity structure (58, 59). We include this module as a minimal, generic mechanism for transforming discrete sensory events into temporally dispersed, heterogeneous mossy-fiber activity with variable onset latencies, as observed experimentally (45, 47, 48), rather than as a claim about the exact biological implementation. The network consists of two neuronal populations: an excitatory (E) population of $N_{E}$ (*N_E_* = 1,600) pyramidal neurons and an inhibitory (I) population of $N_{I}$ ($N_{I}=400$) interneurons (58, 59). The excitatory neurons are functionally arranged along a ring structure representing a continuous feature space.

Both pyramidal neurons and interneurons are modeled as conductance-based leaky integrate-and-fire (LIF) model. The membrane potential $V_{i}\left(t\right)$ of neuron i evolves according to:

[1] $$\tau_{m,P}\frac{dV_{i}\left(t\right)}{\mathrm{dt}}=-\left(V_{i}\left(t\right)-V_{L}\right)-\frac{1}{g_{leak,P}}I_{syn,i}\left(t\right),$$

where P, either E or I, denotes the population type. Here, $\tau_{m,P}$ is the membrane time constant, $V_{L}$ is the leak reversal potential, and $g_{leak,P}$ is the leak conductance. When $V_{i}\left(t\right)$ exceeds the threshold $V_{\mathrm{th}}$, the neuron emits a spike, after which the potential is reset to $V_{\mathrm{reset}}$ and held for a refractory period $\tau_{ref,P}$. $I_{syn,i}\left(t\right)$ represents the total synaptic current received by the neuron. Here, $\begin{array}{c} \tau_{m,E}=20\;\mathrm{ms},\tau_{m,I}=10\;\mathrm{ms},V_{L}=-70\;\mathrm{mV},g_{leak,E}=25\;\mathrm{nS},g_{leak,I}=20\;\mathrm{nS},V_{\mathrm{th}}= \end{array}$-50 mV, $V_{\mathrm{reset}}=-55\;\mathrm{mV}$,$\tau_{ref,E}=2\;\mathrm{ms}$,$\tau_{ref,I}=1\;\mathrm{ms}$. These values were adopted from Ref. 21.

The network features all-to-all recurrent connectivity. Inhibitory projections are spatially uniform and independent of target tuning. Recurrent excitatory currents are mediated by AMPA and NMDA receptors, while inhibitory currents are mediated by GABA receptors. Neurons also receive external excitatory inputs through AMPA receptors, representing both sensory drive and background noise.

For an excitatory neuron:

[2] $$I_{syn,E}=I_{\mathrm{AMPA}}^{E\to E}+I_{\mathrm{NMDA}}^{E\to E}+I_{\mathrm{GABA}}^{I\to E}+I_{\mathrm{ext}}+I_{\mathrm{noise}}+I_{\mathrm{SFA}}.$$

For an inhibitory neuron:

[3] $$I_{syn,I}=I_{\mathrm{AMPA}}^{E\to I}+I_{\mathrm{NMDA}}^{E\to I}+I_{\mathrm{GABA}}^{I\to I}+I_{\mathrm{noise}}.$$

In Eqs. **2** and **3**, the first three terms describe recurrent projections from the excitatory and inhibitory populations; $I_{\mathrm{ext}}$ denotes task-related external input signals, $I_{\mathrm{noise}}$ represents stochastic background noise, and $I_{\mathrm{SFA}}$ denotes spike-frequency–adaptation currents (included only in excitatory neurons).

### Synaptic Dynamics.

Synaptic currents mediated by AMPA and GABA receptors follow:

[4] $$I_{\mathrm{type}}=g_{\mathrm{type}}\cdot {\bar{s}}_{\mathrm{type}}\left(t\right)\cdot \left(V\left(t\right)-V_{rev,type}\right),$$

where type either represents AMPA or GABA, and $V_{rev,type}$ is the corresponding reversal potential.

NMDA currents include a voltage-dependent magnesium block:

[5] $$I_{\mathrm{NMDA}}=g_{\mathrm{NMDA}}\cdot {\bar{s}}_{\mathrm{NMDA}}\left(t\right)\cdot \left(V\left(t\right)-V_{E}\right)\cdot B\left(V\right),$$

[6] $$B\left(V\right)=\frac{1}{1+\frac{1}{b}\text{exp}\left(-aV\left(t\right)\right)}.$$

Spike-frequency adaptation in excitatory neurons is modeled as

[7] $$I_{\mathrm{SFA}}=g_{\mathrm{SFA}}\cdot w_{\mathrm{SFA}}\left(t\right)\cdot \left(V\left(t\right)-V_{K}\right),$$

[8] $$\frac{dw_{\mathrm{SFA}}}{\mathrm{dt}}=-\frac{w_{\mathrm{SFA}}}{\tau_{w}}+b_{\mathrm{SFA}}\sum_{k}\delta \left(t-t_{k}\right).$$

Here, $g_{AMPA,E}=150\;\mathrm{nS}$, $g_{AMPA,I}=300\;\mathrm{nS}$, $g_{NMDA,E}=400\;\mathrm{nS}$, $g_{NMDA,I}=100\;\mathrm{nS}$, $V_{rev,E}=0\;\mathrm{mV}$, $V_{rev,I}=-70\;\mathrm{mV}$, $a=0.062\;{\text{mV}}^{-1}$, b=3.57, and *V_K_* = −80 mV. The AMPA, NMDA, and reversal-potential parameters were adapted from Ref. 21. The spike-frequency adaptation parameters, $g_{\mathrm{SFA}}=100\;\mathrm{nS}$, $\tau_{w}=80\;\mathrm{ms},$ and $b_{\mathrm{SFA}}=0.1$ were chosen to satisfy explicit dynamical constraints, stable continuous-attractor activity, and temporally dispersed responses resembling experimentally observed heterogeneous mossy-fiber latencies (45, 47, 48).

### Synaptic Gating Variables.

The synaptic gating variable $\bar{s}\left(t\right)$ represents the fraction of open channels. For recurrent connections:

[9] $$\bar{s}\left(t\right)=\sum_{j}w_{\mathrm{ij}}s_{j}\left(t\right),$$

where $w_{\mathrm{ij}}$ is the synaptic weight from presynaptic neuron j to postsynaptic neuron i, and $s_{j}\left(t\right)$ is the gating variable driven by presynaptic spikes.

For external inputs, $\bar{s_{i}}=s_{i}$, determined independently by each neuron’s input pulse sequence.

For AMPA and GABA receptors, $s_{X}\left(t\right)$ evolves as

[10] $$\frac{ds_{X}\left(t\right)}{\mathrm{dt}}=-\frac{s_{X}}{\tau_{X}}+\sum_{k}\delta \left(t-t_{k}\right),$$

where X represents AMPA or GABA, and $t_{k}$ denotes the presynaptic spike times.

NMDA receptor kinetics follow a two-stage process:

[11] $$\frac{ds_{\mathrm{NMDA}}}{\mathrm{dt}}=-\frac{s_{\mathrm{NMDA}}}{\tau_{\mathrm{NMDA}}}+\alpha x\left(1-s_{\mathrm{NMDA}}\right),$$

[12] $$\frac{\mathrm{dx}}{\mathrm{dt}}=-\frac{x}{\tau_{x}}+\sum_{k}\delta \left(t-t_{k}\right).$$

Here, $\tau_{\mathrm{AMPA}}=2\;\mathrm{ms}$, $\tau_{\mathrm{GABA}}=5\;\mathrm{ms}$, $\tau_{\mathrm{NMDA}}=100\;\mathrm{ms}$, $\tau_{x}=2\;\mathrm{ms}$, α=0.5 kHz. These values were adopted from Ref. 21.

### Background Inputs and Connectivity.

All neurons receive independent Poisson background inputs via AMPA receptors, with firing rate $\nu_{\mathrm{background}}$.

The maximal conductances for background noises targeting excitatory and inhibitory neurons are $g^{background\to E}$ and $g^{background\to I}$, respectively.

Recurrent connection strength between two excitatory neurons depends on the difference between their preferred angles $\theta_{i}$ and $\theta_{j}$:

[13] $$w_{\mathrm{ij}}=W\left(\Delta \theta \right)=J_{-}+\left(J_{+}-J_{-}\right)\;\text{exp}\;\left(-\frac{\Delta \theta^{2}}{2\sigma_{w}^{2}}\right),$$

where $J_{+}$ represents the peak synaptic strength, $J_{-}$ is the baseline connectivity, and $\sigma_{w}$ determines the spatial width of the connectivity profile. Here, $\nu_{\mathrm{background}}=100\;\mathrm{Hz}$, $g^{background\to E}=1\;\mathrm{nS}$, $g^{background\to I}=1.62\;\mathrm{nS}$, $J_{+}=35$, $J_{-}=0$, $\sigma_{w}=2.4$. Together with the spike-frequency adaptation parameters in Eqs. **7** and **8**, these values were selected to yield stable continuous-attractor dynamics while producing temporally dispersed population responses with heterogeneous onset latencies.

### Cortical Decision-Making Module.

The cortical decision-making module was adapted from the cortical network model proposed by Wang (21). This module was included to examine the benefits of combining two degenerate decision-making modules. It integrates evidence derived either from Purkinje neurons (in the cortico-cerebello-cortical model) or from the cortical continuous attractor network (in the cortico-cortical model).

The network consists of $N_{E}$ excitatory pyramidal neurons and $N_{I}$ inhibitory interneurons. Neuronal and synaptic dynamics follow the continuous attractor model described previously, with the following modifications to implement decision-making functionality.

The excitatory population is divided into three distinct subpopulations:

a. Non-selective population ($N_{0})$—responds only to background noise.
b. Selective population 1 ${(N}_{1})$—encodes the left decision, receiving input from Purkinje neurons or attractor neurons representing left-side cues.
c. Selective population 2 ${(N}_{2})$—encodes the right decision, receiving input from Purkinje neurons or attractor neurons representing right-side cues.

The size of the selective populations is determined by the fraction of selective neurons, $f_{\mathrm{sel}}$, such that $N_{1}=N_{2}=f_{\mathrm{sel}}N_{E}.$

All neurons receive global inhibition from the inhibitory pool, while excitatory connectivity is structured to promote competition. Synaptic weights between excitatory neurons, $w_{\mathrm{ij}}$, depend on population identity:

Within the same selective population (recurrent excitation), $w_{+}=w_{p}$.

Between different selective populations, $w_{-}=\left(1-f_{\mathrm{sel}}w_{p}\right)/\left(1-f_{\mathrm{sel}}\right)$.

All other excitatory and inhibitory connections are set to unity (w=1).

Each neuron receives independent Poisson background noise via AMPA receptors at a rate of $\nu_{\mathrm{ext}}$. Evidence signals originating from the cerebellum or the cortical continuous attractor module are modeled as external AMPA currents delivered to the corresponding selective populations. Here, *N_E_* = 1,600, $N_{I}=400$, $f_{\mathrm{sel}}=0.15$, $w_{p}=1.7$, $\nu_{\mathrm{ext}}=2.4\;\mathrm{kHz}$. These values were adopted from Ref.^21^.

### Cerebellar Model.

The cerebellar circuit was modeled largely as a feedforward architecture. The network consists of three layers: 1) an input layer of mossy fibers, 2) a granule cell layer containing granule and Golgi cells, and 3) an output layer composed of Purkinje neurons.

Except in Fig. 2, where each sensory stimulus is represented by a burst of spikes, the cerebellar model receives input from excitatory populations of the cortical continuous attractor module. The axon terminals of cortical neurons are modeled as mossy fibers, with a total of 60 fibers.

The granule cell layer contains $N_{\mathrm{gc}}$ excitatory granule cells and $N_{\mathrm{Golgi}}$ inhibitory Golgi cells. Granule cell membrane dynamics follow the adaptive quadratic integrate-and-fire (AdQIF) model:

[14] $$C_{\mathrm{gc}}\frac{dV\left(t\right)}{\mathrm{dt}}=k\left(V-V_{r}\right)\left(V-V_{t}\right)-u+I_{\mathrm{syn}}^{\mathrm{gc}},$$

[15] $$\frac{du\left(t\right)}{\mathrm{dt}}=a_{\mathrm{gc}}\left(b_{\mathrm{gc}}\left(V-V_{r}\right)-u\right),$$

where V is the membrane potential and u the adaptation variable. $C_{\mathrm{gc}}$ is the membrane capacitance, k a scaling factor, $V_{r}$ the resting potential, and $V_{t}$ the threshold parameter. Adaptation dynamics are controlled by the time constant $a_{\mathrm{gc}}$ and sensitivity parameter $b_{\mathrm{gc}}$. When V reaches $V_{\mathrm{peak}}$, the potential is reset to $c_{\mathrm{reset}}$ and u is incremented by $d_{\mathrm{reset}}$.

The total synaptic current is

[16] $$I_{\mathrm{syn}}^{\mathrm{gc}}=I_{\mathrm{AMPA}}^{mf\to gc}+I_{\mathrm{GABA}}^{goc\to gc}+I_{\mathrm{noise}}.$$

AMPA, GABA, and background Poisson noise currents are computed as in the cortical model.

Golgi cells provide feedback inhibition to regulate granule cell gain and temporal dynamics. They are modeled as leaky integrate-and-fire (LIF) neurons:

[17] $$C_{\mathrm{Golgi}}\frac{dV\left(t\right)}{\mathrm{dt}}=g_{l,Golgi}\left(E_{l,Golgi}-V\right)+I_{\mathrm{syn}}^{\mathrm{Golgi}}\left(t\right),$$

where $C_{\mathrm{Golgi}}$ is the membrane capacitance and $g_{l,Golgi}$ is the leak conductance. The total synaptic current is

[18] $$I_{\mathrm{syn}}^{\mathrm{Golgi}}\left(t\right)=I_{\mathrm{AMPA}}^{gc\to Golgi}+I_{\mathrm{noise}}.$$

The output layer comprises $N_{\mathrm{PN}}$ Purkinje neurons (half per side). Each Purkinje neuron is modeled using the adaptive exponential integrate-and-fire (AdEx) formalism with spike-triggered adaptation and colored noise, as in Ref. 52:

[19] $$C_{\mathrm{PN}}\frac{dV\left(t\right)}{\mathrm{dt}}=g_{L}\Delta_{T}\text{exp}\left(\frac{V-V_{T}}{\Delta_{T}}\right)-g_{L}\left(V-E_{L}\right)-w+I_{\mathrm{hold}}+I_{\mathrm{OU}}+I_{\mathrm{syn}}^{\mathrm{PN}},$$

where $C_{\mathrm{PN}}$ is the membrane capacitance, $g_{L}$ the leak conductance, and $E_{L}$ the leak reversal potential. The exponential term models the rapid depolarization during spike generation, with slope factor $\Delta_{T}$ and threshold $V_{T}$.

The adaptation variable w follows:

[20] $$\tau_{w}\frac{dw\left(t\right)}{\mathrm{dt}}=a_{\mathrm{PN}}\left(V-E_{L}\right)-w.$$

Once V reaches 60 mV, a spike is emitted. V is then reset to $V_{\mathrm{reset}}$, and w is incremented by $b_{\mathrm{PN}}$.The Purkinje neuron excitability class was controlled by varying two dimensionless parameters, $A=a_{\mathrm{PN}}/g_{L}$ and $T=\tau_{w}/\tau_{m}=\tau_{w}g_{L}/C_{\mathrm{PN}}$ (81). Here, $\tau_{m}=C_{\mathrm{PN}}/g_{L}$ denotes the membrane time constant. For the type-I regime, we set A=0.12 (corresponding to $a_{\mathrm{PN}}=1\;\mathrm{nS}$ and $g_{L}=8.47\;\mathrm{nS})$ and T=0.66 (corresponding to $\tau_{w}=20.76\;\mathrm{ms}$, $g_{L}=8.47\;\mathrm{nS}$, and $C_{\mathrm{PN}}=268\;\mathrm{pF}$). For the type-II regime, we set A=4.46 (corresponding to $a_{\mathrm{PN}}=37.79\;\mathrm{nS}$) and $g_{L}=8.47\;\mathrm{nS}$, while keeping T=0.66 with the same $\tau_{w}$,$g_{L}$, and $C_{\mathrm{PN}}$.

For computational tractability, we used the single-compartment AdEx model rather than a multicompartment conductance-based Purkinje neuron model (57) and therefore did not explicitly represent the specific ionic currents that biophysically underlie type-II dynamics. Accordingly, the “type-I” regime is included as a matched computational control to isolate the contribution of hysteretic type-II-like dynamics, not to suggest a distinct biological class of type-I Purkinje neurons.

Intrinsic background activity is modeled using Ornstein–Uhlenbeck (OU) noise:

[21] $$I_{\mathrm{OU}}=\sigma \cdot \xi \left(t\right),\;\frac{d\xi }{\mathrm{dt}}=-\frac{\xi }{\tau_{\mathrm{corr}}}+\sqrt{\frac{2}{\tau_{\mathrm{corr}}}}\eta \left(t\right),$$

where σ is the noise SD, $\tau_{\mathrm{corr}}$ the correlation time constant, and $\eta \left(t\right)$ Gaussian white noise. A constant holding current $I_{\mathrm{hold}}$ modulates baseline excitability. For simplicity, cerebellar nuclei neurons are not modeled explicitly; their function is merged into the Purkinje neuron population, which acts as an effective output to downstream cortical circuits.

Each granule cell receives excitatory input from four randomly selected mossy fibers. Granule cell and Golgi cells form a feedback inhibition loop:

1. granule → Golgi connections (AMPA): $p_{gc\to Golgi}=0.1$
2. Golgi → granule connections (GABA): $p_{Golgi\to gc}=0.5$

This loop regulates granule cell gain and temporal sparsity. Granule cells project to Purkinje neurons via parallel fibers. The synaptic weights $w_{PF-PN}$ are plastic, following a reward-dependent plasticity framework (82) with an anti-Hebbian rule (83):

$$\begin{array}{c} \mathrm{Pre}-\mathrm{before}-\mathrm{Post}:c\leftarrow c+A_{\mathrm{pre}}\cdot \text{exp}\;\left(-\Delta t/\tau_{\mathrm{pre}}\right), \end{array}$$

[22] $$\text{Post}-\text{before}-\text{Pre}:c\leftarrow c+A_{\mathrm{post}}\cdot \text{exp}\;\left(-\Delta t/\tau_{\mathrm{post}}\right),$$

$$w_{PF-PN}\leftarrow w_{PF-PN}+\eta \cdot c,$$

where η is the learning rate, and $\tau_{\mathrm{pre}}$ and $\tau_{\mathrm{post}}$ define the temporal windows for plasticity. c denotes the synaptic eligibility trace. The training procedure was as follows. During the cue period, a punishment signal (a negative reward or reinforcement signal) was delivered if the model made an incorrect decision. Training was continued until the synaptic weights reached a steady state. The synaptic weights $w_{PF-PN}$ were updated only when the punishment signal was delivered after the end of the cue period. The resulting evolution of synaptic weights and model performance during training is shown in *SI Appendix*, Fig. S10. At the start of training, granule cells activated by either left- or right-side stimuli have equal synaptic strengths onto both left- and right-selective Purkinje populations, so the decision output is effectively random. During training, when an incorrect choice is followed by a punishment/error signal, the elevated activity of the Purkinje population encoding the chosen (incorrect) side leads to selective depression of synapses from the currently activated granule cells onto that contralateral (cross) Purkinje population, while synapses onto the ipsilateral Purkinje population are largely unchanged. After training reaches steady state, stimulus-activated granule cells form substantially stronger connections to ipsilateral Purkinje neurons than to contralateral Purkinje neurons, thereby improving decision accuracy.

Although we did not explicitly model climbing fibers or climbing-fiber–evoked complex spikes as dynamical components, their functional role is incorporated into the training procedure. This choice is motivated by evidence that reward prediction and negative reward signals can be conveyed by climbing-fiber–evoked complex spikes (9, 84–86). Incorporating a more biophysically detailed plasticity rule with dynamically regulated LTD and LTP (86, 87) will be an important direction for future work.

Here, $C_{\mathrm{gc}}=100\;\mathrm{pF}$, $V_{r}=-60\;\mathrm{mV}$, $V_{t}=-40\;\mathrm{mV}$, $k=0.7\;\mathrm{pA}/({\text{mV}}^{2})$, $a_{\mathrm{gc}}=0.03\;{\text{ms}}^{-1}$, $b_{\mathrm{gc}}=-2\;\mathrm{nS}$, $C_{\mathrm{Golgi}}=50\;\mathrm{pF}$, $g_{l,Golgi}=5\;\mathrm{nS}$, $E_{l,Golgi}=$-65 mV, $N_{\mathrm{PN}}=400$, $C_{\mathrm{PN}}=268\;\mathrm{pF}$, $g_{L}=8.47\;\mathrm{nS}$, $\Delta_{T}=0.85\;\mathrm{mV}$, $V_{T}=$-53.23 mV, $E_{L}=-51.31\;\mathrm{mV}$, $\tau_{w}=20.76\;\mathrm{ms}$, $a_{\mathrm{PN}}=37.79\;\mathrm{nS}$, $b_{\mathrm{PN}}=$441.12 pA, $V_{\mathrm{reset}}=-60.35\;\mathrm{mV}$, σ=40 pA, $\tau_{\mathrm{corr}}=2\;\mathrm{ms}$, $I_{\mathrm{hold}}=-140\;\mathrm{pA}$, $A_{\mathrm{pre}}=A_{\mathrm{post}}=-0.01$. The granule-cell and Golgi-cell parameters were adopted from Ref. 83. The Purkinje-neuron parameters were selected to exhibit a stable type-II excitability regime (52–54).

### Sensitivity to Mutual Inhibition Between Purkinje Neurons.

Mutual inhibition between Purkinje neurons is critical for evidence competition. Excessively high inhibition conductance destabilizes baseline firing, producing spontaneous bistability, which is biologically unrealistic (60). A conductance value is considered biologically plausible if the firing-rate difference between the two Purkinje neuron populations remains below 2 Hz after 6,500 ms of simulation without external stimulation.

The fraction of completed trials was defined as the ratio of valid trials to total trials. To handle undecided trials conservatively, we followed the standard practice of random assignment to left or right decisions while also reporting the fraction of completed trials as an independent metric.

### t-SNE Visualization of Granule Cell Population Activity.

To visualize high-dimensional granule cell activity across sparsity levels, we applied t-Distributed Stochastic Neighbor Embedding (t-SNE). Spike trains from *N* = 2,000 granule cells were converted into population state vectors using non-overlapping 20 ms bins. Each vector contained spike counts across all recorded neurons. State vectors from left- and right-dominant conditions were merged for joint analysis.

t-SNE was implemented using Scikit-learn with the following parameters:

$$\begin{array}{c} \text{Components}=3;\;\text{Perplexity}\;=\;15;\;\text{Initialization}=\text{PCA};\;\\ \text{Distance}\;\text{metric}=\text{Euclidean}. \end{array}$$

This configuration balances local/global structure preservation and ensures stable manifold visualization.

### Population Firing Rate.

Instantaneous population firing rates were computed using a 100 ms sliding window moving in 2 ms steps. Within each window, total spikes were divided by the number of neurons and the window duration to obtain population firing rates.

### Simulations.

All simulations were performed on a Linux workstation. The coupled differential equations describing neuronal and synaptic dynamics were numerically integrated using a modified second-order Runge–Kutta (RK2) algorithm with a time step of 0.02 ms.

Decision-making performance was evaluated as the average percentage of correct choices across n=200 trials for each parameter set and left–right stimulus condition.

## Supplementary Material

Appendix 01 (PDF)

## Data Availability

The simulation code used in this study is publicly available at GitHub (88).
