# Supplementary material for: Cerebellar microcircuits enable robust evidence-based decisions through cortico–cerebellar coupling
Source: Proc Natl Acad Sci U S A. 2026 Aug 27;123(35):e2616911123. doi: 10.1073/pnas.2616911123 (PMC13534631; doi:10.1073/pnas.2616911123)
Supplement: Supplementary file 1 [file pnas.2616911123.sapp.pdf]

Supporting Information for

Cerebellar microcircuits enable robust evidence-based decisions through cortico–cerebellar coupling

Yeyao Bao, Liao Yu, Liangfu Lu, Zhuoqin Yang, Yunliang Zang

Yunliang Zang
Email: yunliangzang@tju.edu.cn

This PDF file includes:

Figures S1 to S10

Supplementary Figures

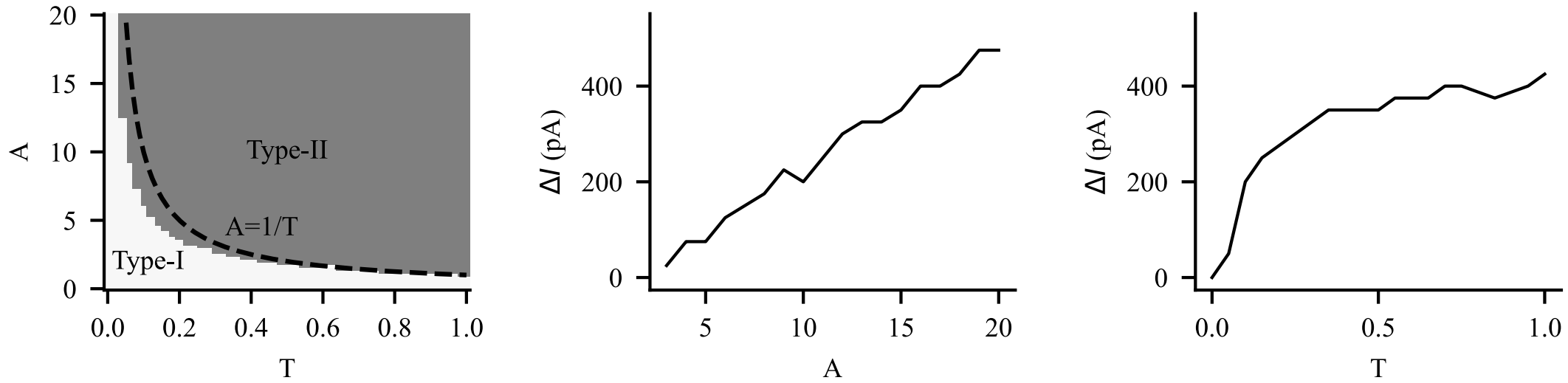

Fig. S1. Parameter map of temporal response properties in the Purkinje-neuron model

Left: excitability regimes of the model in the (A, T) Parameter space. The dashed line denotes the theoretically derived boundary between type-I and type-II excitability (1). Middle/right: dependence of ΔI on A and T. Here $\Delta I = I_{on} - I_{off}$, where I_{on} is the firing onset current during an ascending ramp and I_{off} is the firing offset current during a descending ramp. Parameters are $A = a_{PN}/g_L$ and $T = \tau_w/\tau_{m_L} = (\tau_w g_L)/C_{PN}$.

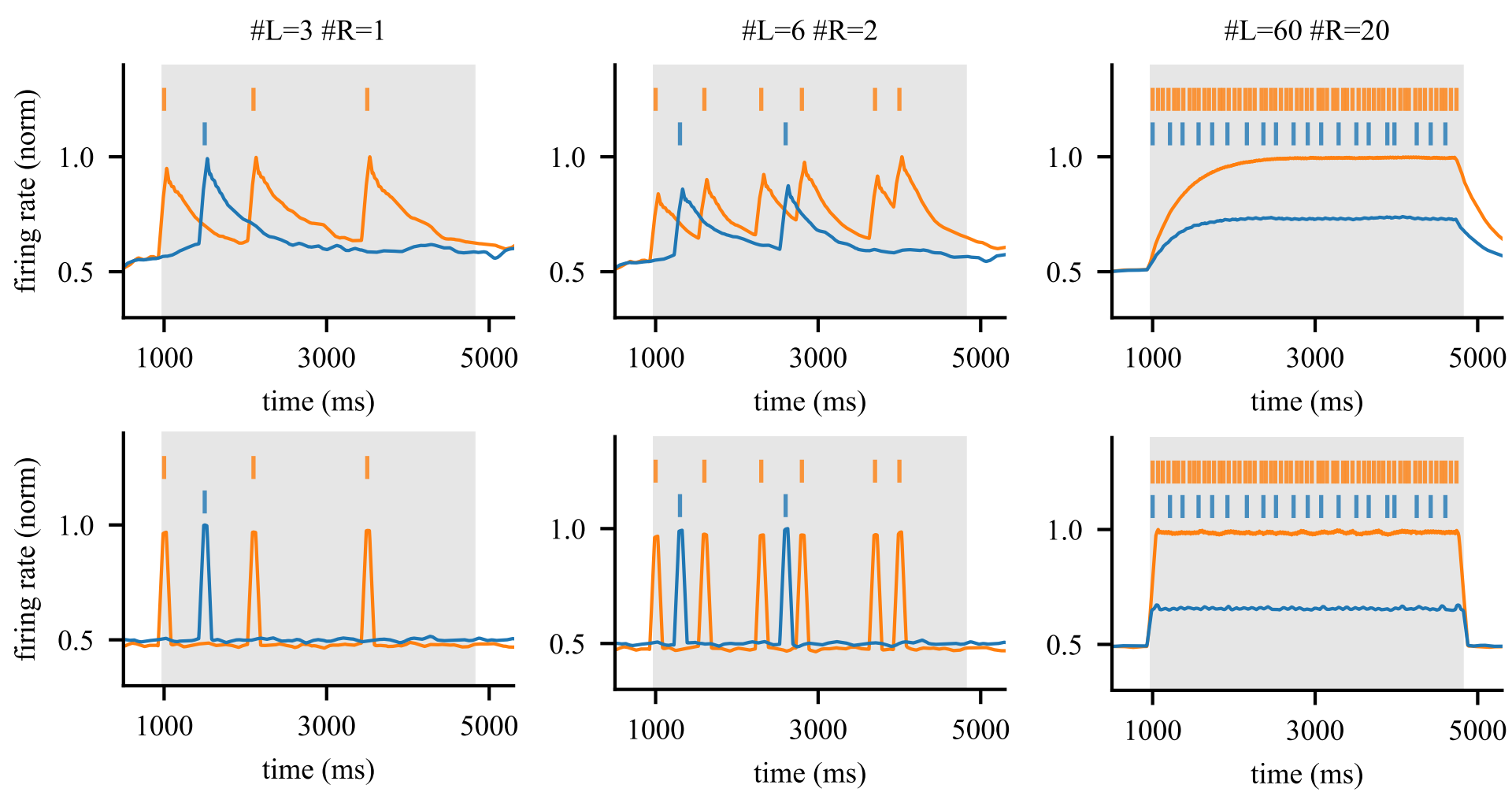

Fig. S2. Purkinje neuron firing rate accumulation dynamics (type II, top; type I, bottom) without mutual inhibition across increasing stimulus frequencies

Colored vertical lines mark individual stimulus, while colored traces show the corresponding firing-rate responses of Purkinje neurons on each side.

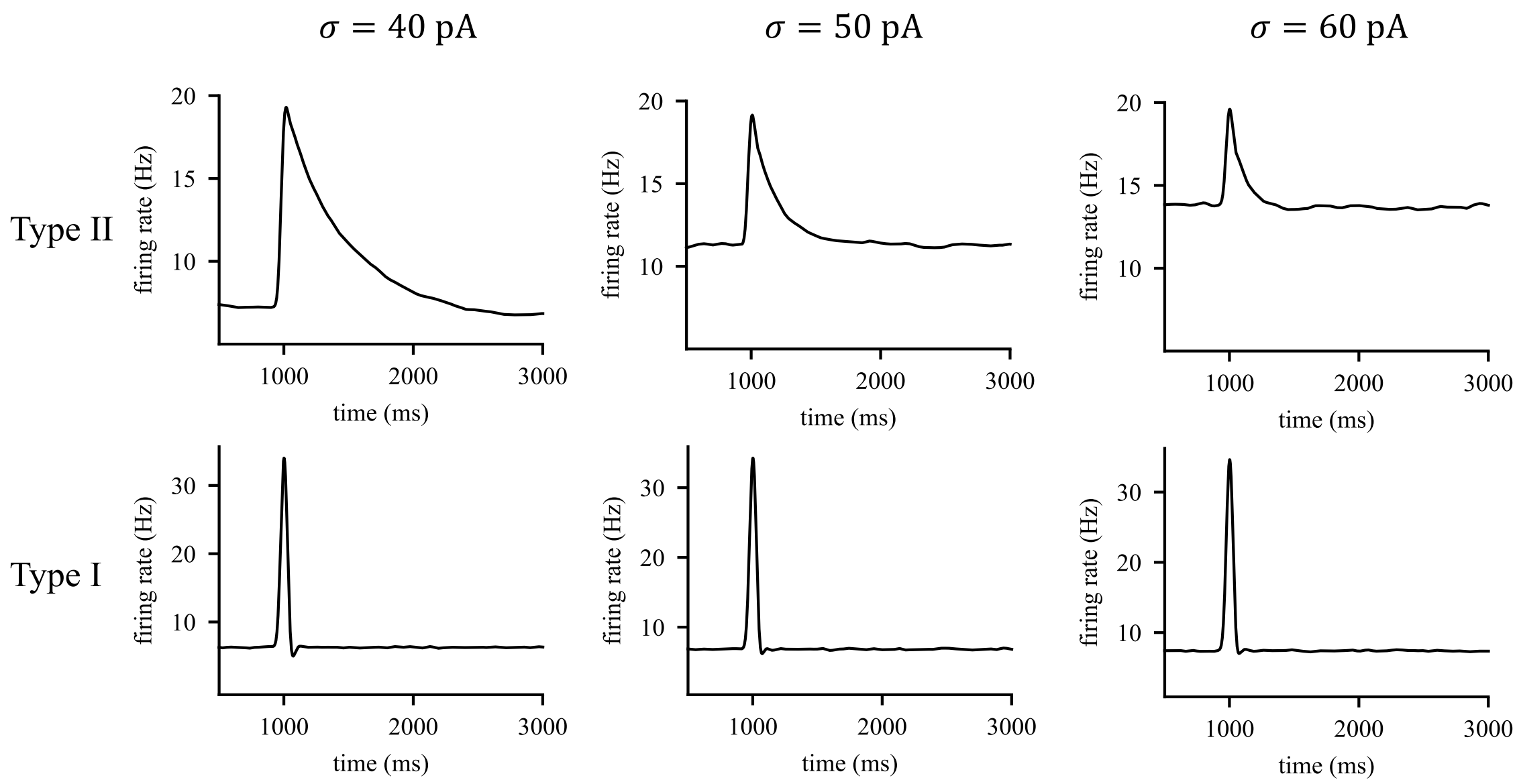

Fig. S3. Example population firing rates of Purkinje neurons at different noise variances
Population firing-rate responses are shown for the Type II model (top) and Type I model (bottom) under varying levels of input noise. The holding current $I_{hold} = -140\ pA$.

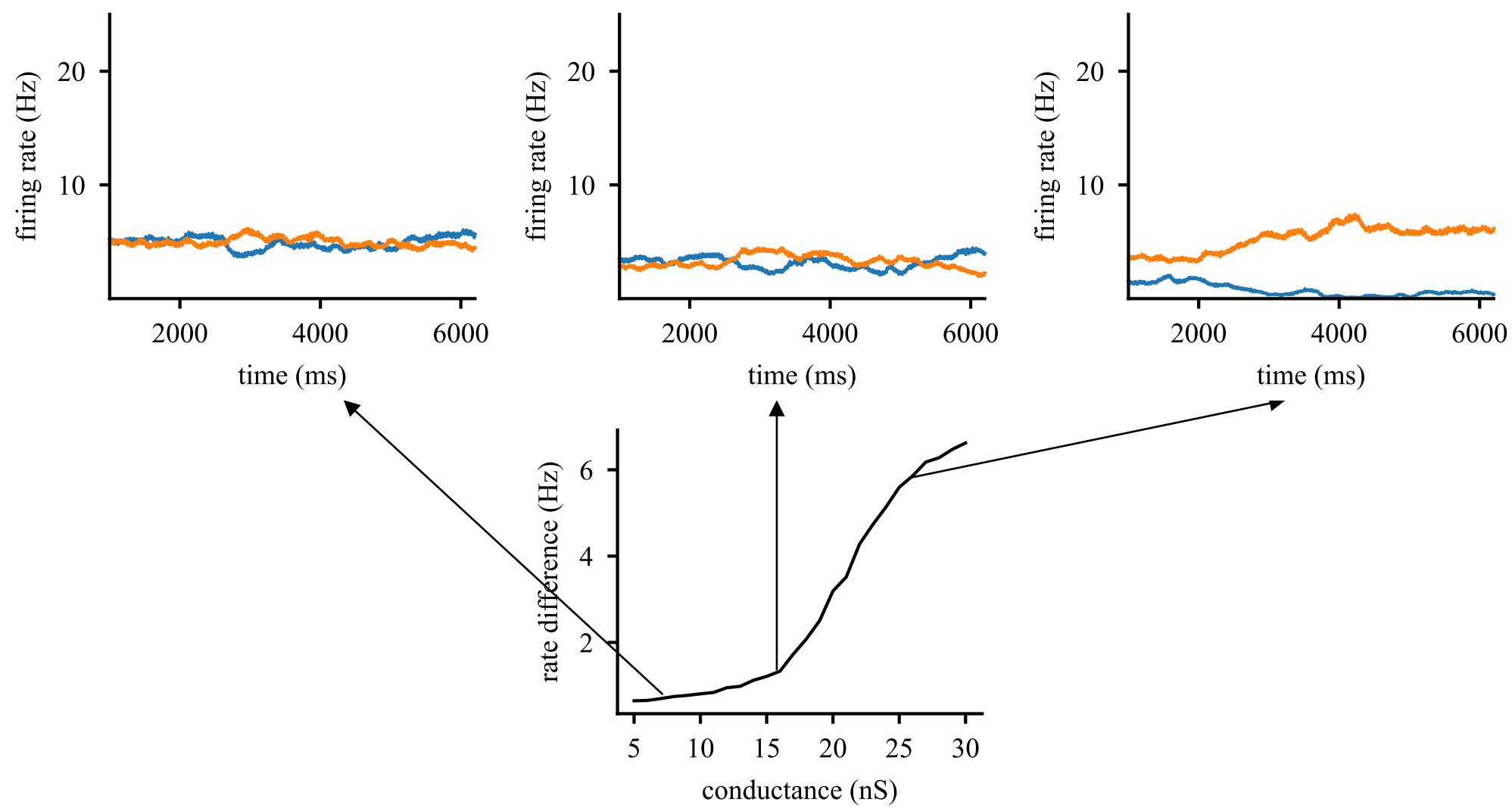

Fig. S4. Instability of Purkinje neuron spontaneous firing dynamics at large mutual inhibition strengths

Increasing mutual inhibition beyond a critical threshold leads to unstable spontaneous firing behaviors in Purkinje neurons.

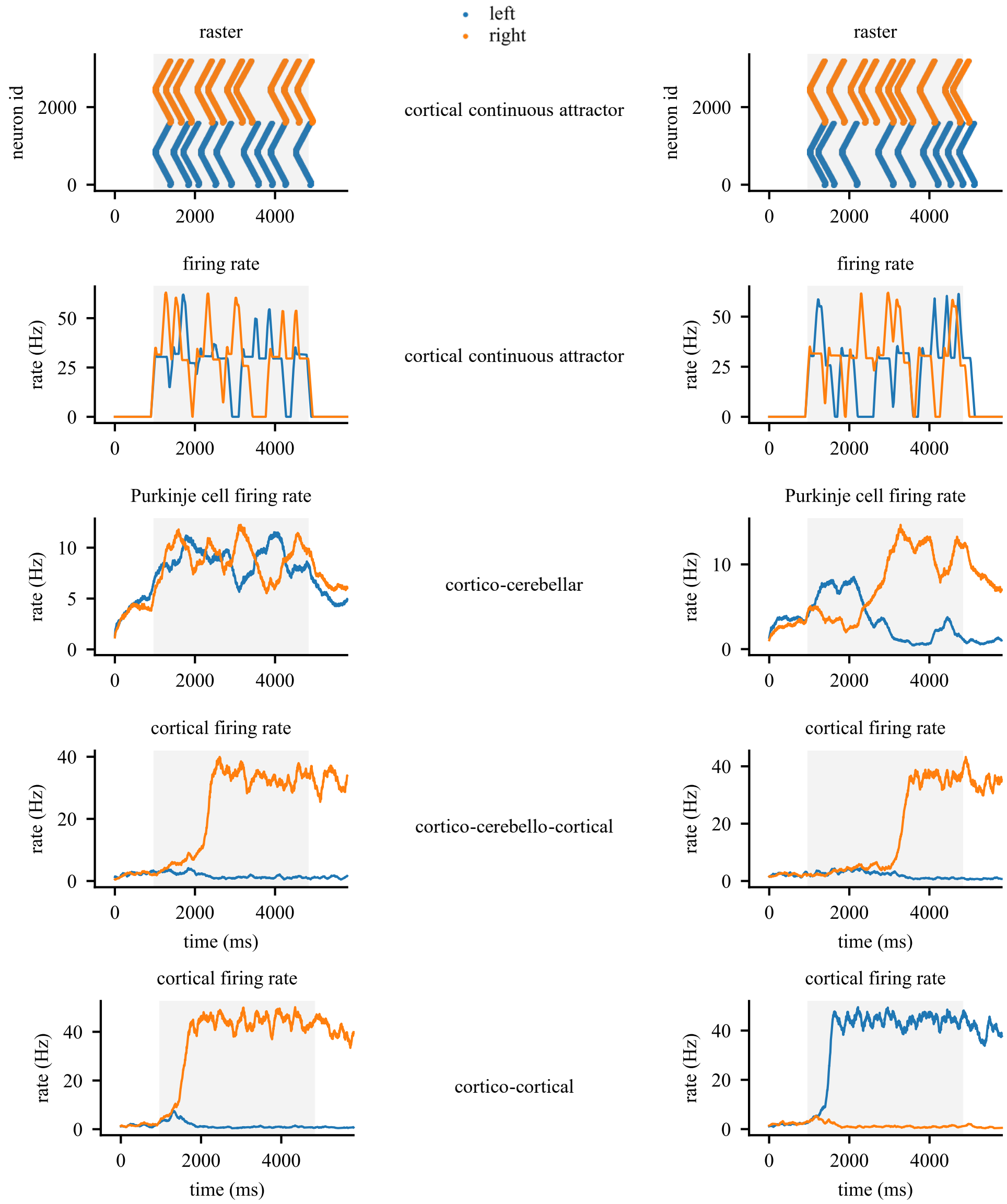

Fig. S5. Example circuit dynamics illustrating complementary roles of cerebellar and cortical decision modules

From top to bottom: spike raster in the cortical continuous-attractor module, corresponding population firing rates, Purkinje-neuron population firing rates in the cortico–cerebellar model, cortical population firing rates in the cortico–cerebello–cortical model, and cortical population firing rates in the cortico–cortical model. In both columns, evidence favors the left choice. In the left example, the cortico–cerebellar model fails to reach a categorical decision, whereas both the cortico–cortical and cortico–cerebello–cortical models choose correctly. In the right example, the cortico–cortical model chooses incorrectly due to a primacy bias, whereas both the cortico–cerebellar and cortico–cerebello–cortical models choose correctly.

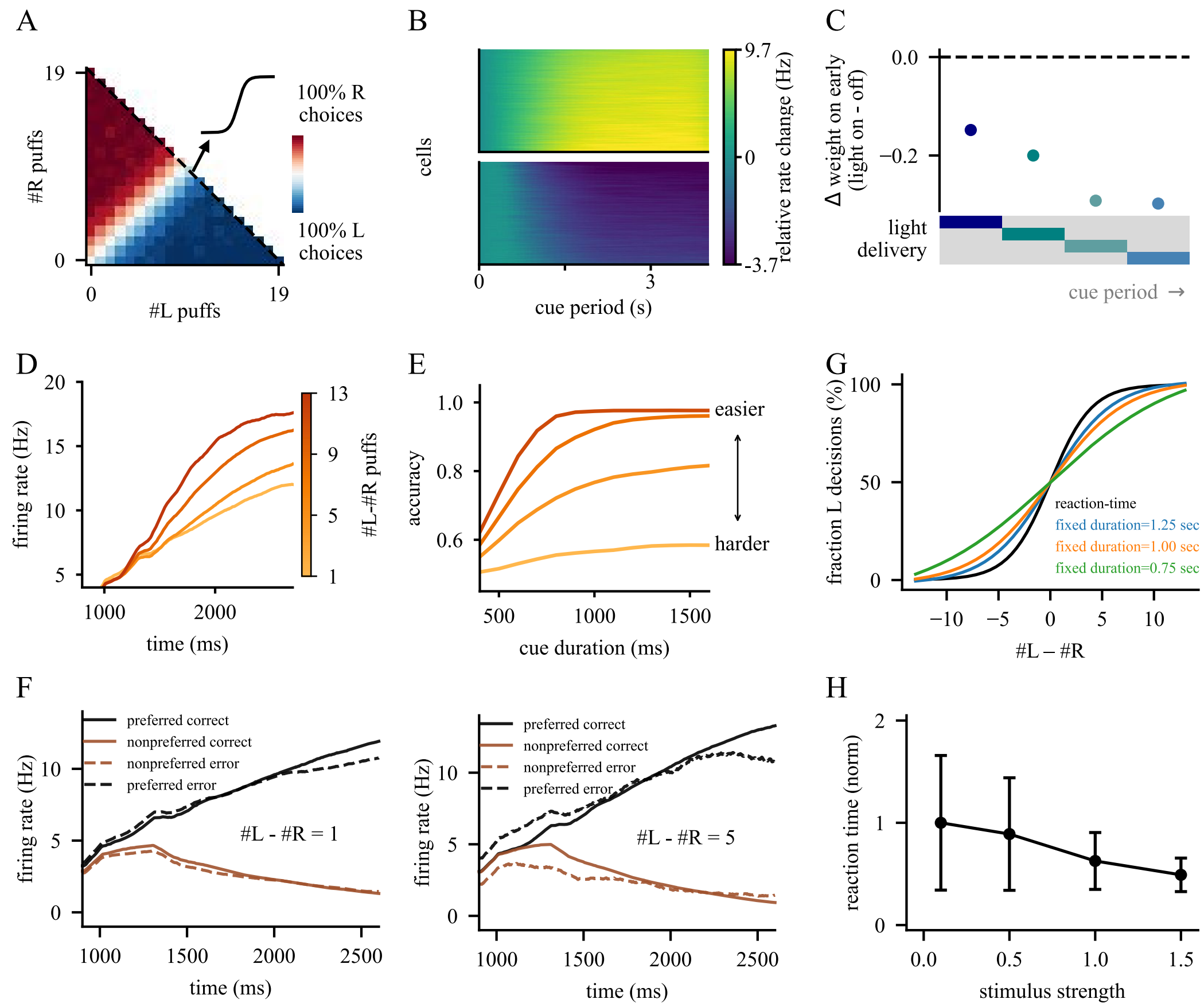

Fig. S6. Evidence-accumulation dynamics can also be captured by the cortico–cerebellar model

(A) Choice probability as a function of left and right puff counts. (B) Cue-period activity of winning (top) and losing (bottom) Purkinje neuron populations. (C) Weight change following cue-period Purkinje neuron perturbations applied at different times. (D) Winning-population Purkinje neuron trajectories across evidence differences. (E) Psychometric curves across cue durations and difficulty levels. (F) Population trajectories for correct and error trials across stimulus differences. (G) Psychometric curves comparing different fixed cue durations with the reaction-time paradigm. (H) Mean reaction time changes versus stimulus strength. Panels A–F: fixed-time; panel H: reaction-time; panel G: both. Compared with Figure 7, panels B and D are identical; all other panels differ.

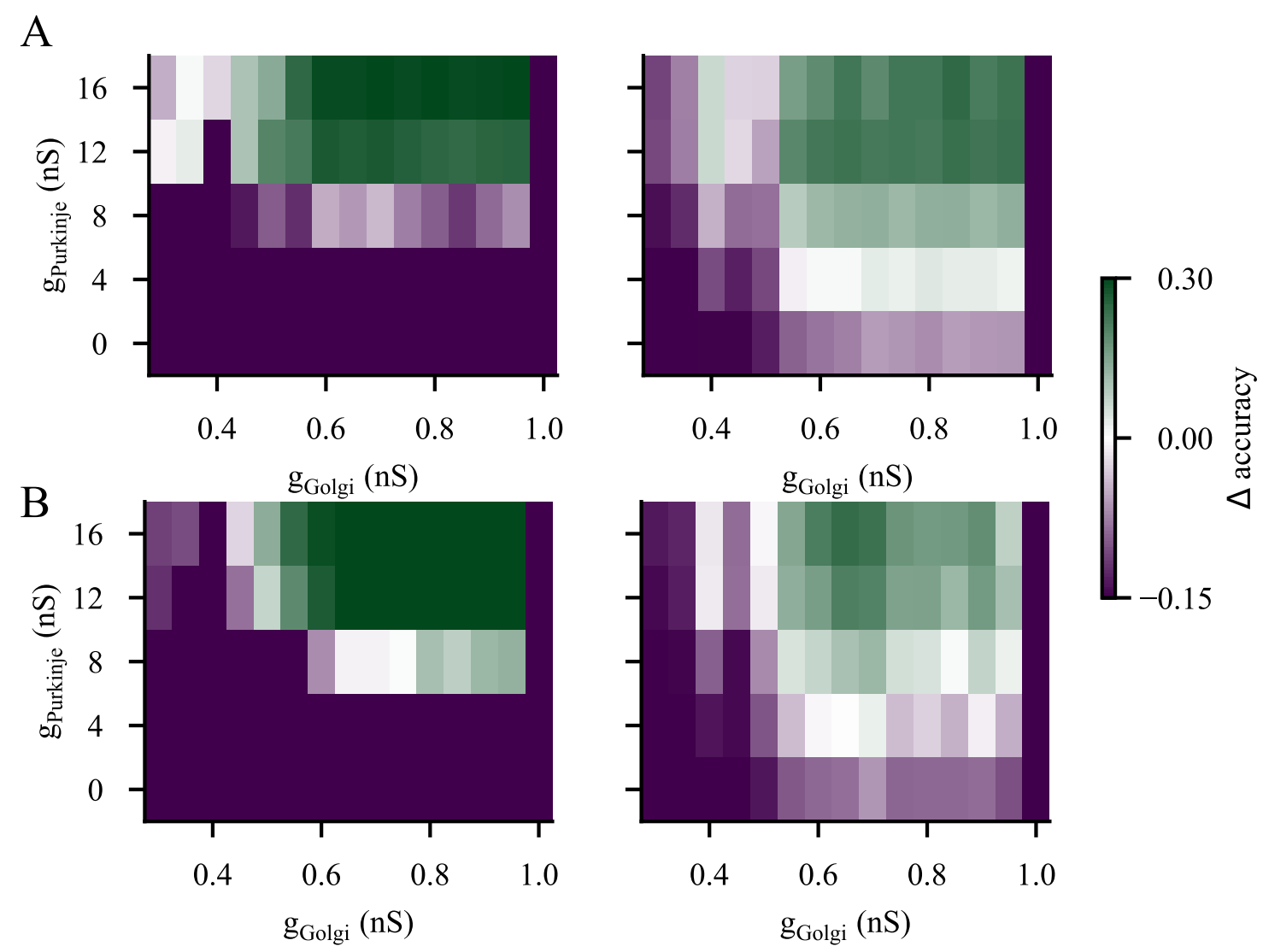

Fig. S7. Accuracy-gain heatmaps as a function of Golgi cell and Purkinje neuron inhibition strengths at overlap ratios of 40% (A) and 60% (B)

Heatmaps show the relative improvement in decision accuracy for different inhibitory strengths. Left panels depict cortico-cerebellar minus cortico-cortical performance; right panels depict cortico-cerebello-cortical minus cortico-cortical performance.

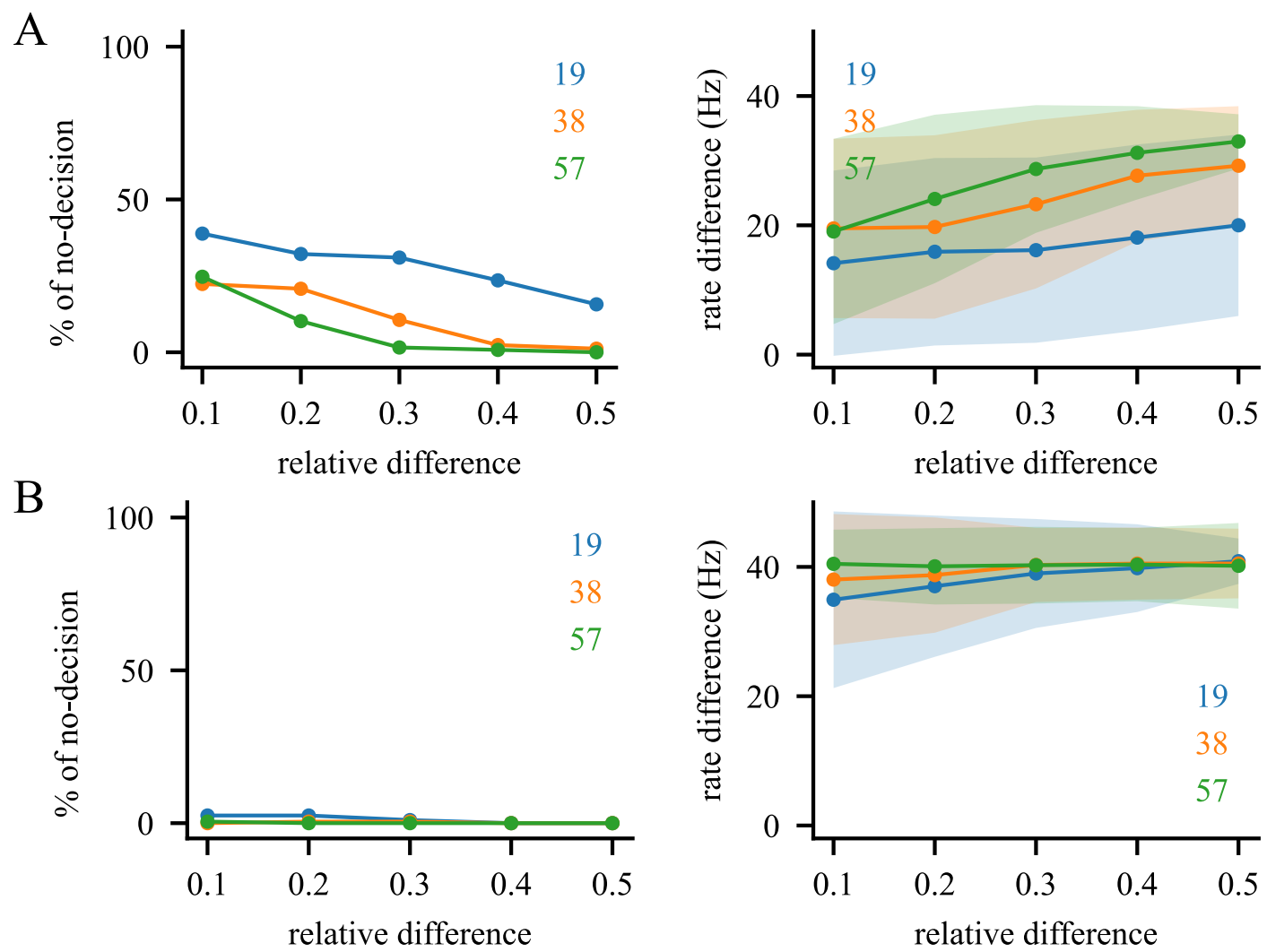

Fig. S8. Cortical preprocessing enhances evidence accumulation in the cortico-cerebello-cortical Model

(A) Proportion of undecided trials (left) and firing-rate difference between opposing cortical populations at the end of the cue period in response to direct sensory-triggered spike bursts. (B) Same analysis as in (A), but for cortical-preprocessed inputs, showing improved decision formation and reduced indecision. Colored numbers indicate total evidence across both sides.

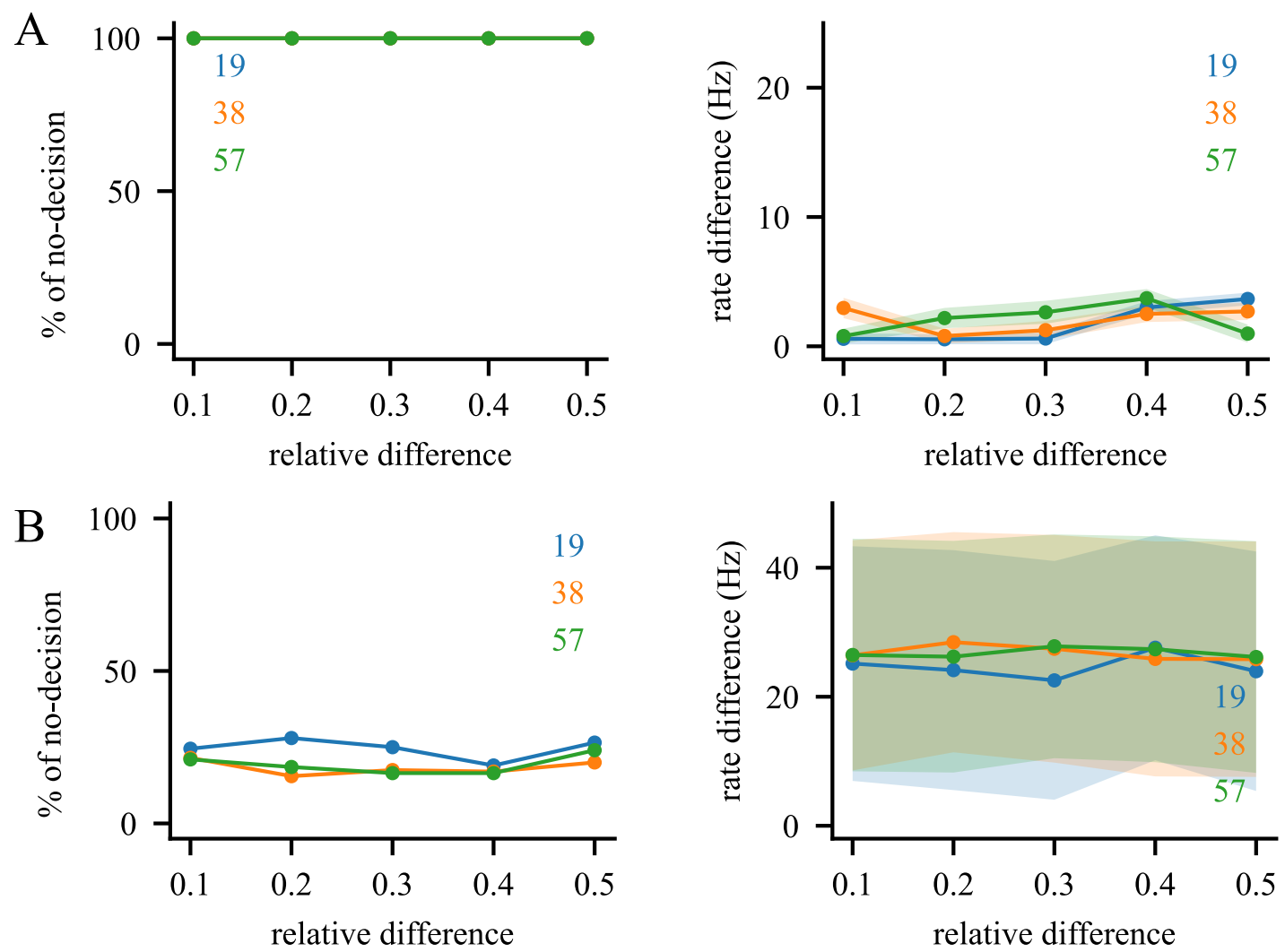

Fig. S9. Firing-rate hysteresis is required for evidence accumulation across model configurations

(A) Cortico-cerebellar model with type-I Purkinje neurons in the cerebellar module. Left: proportion of undecided trials. Right: firing-rate difference between the two cortical populations at the end of the cue period. (B) Cortico-cerebello-cortical model with type-I Purkinje neurons in the cerebellar module. Left: proportion of undecided trials. Right: firing-rate difference between the two cortical populations at the end of the cue period.

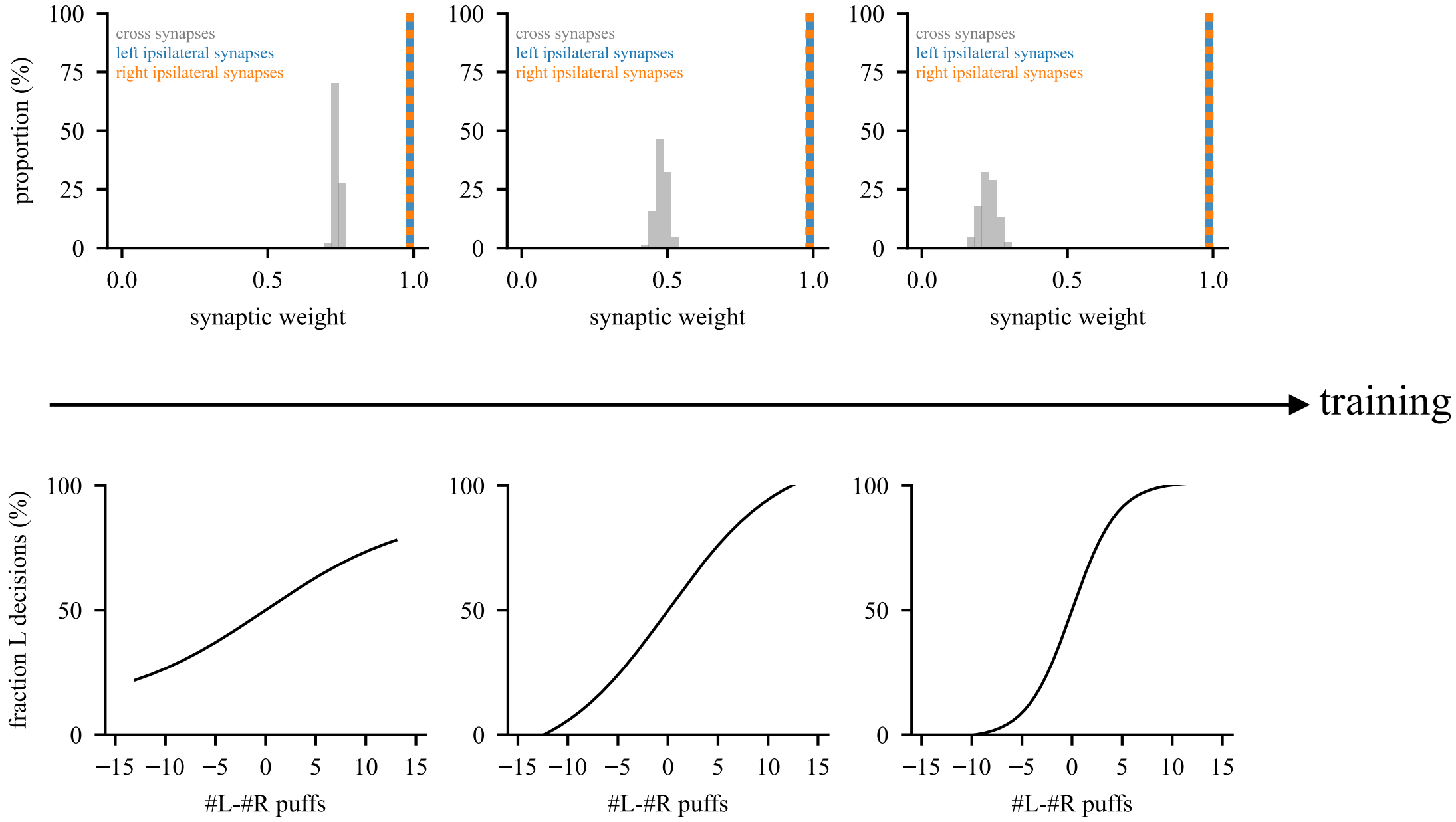

Fig. S10. Synaptic-weight changes and performance during training

Top: evolution of synaptic weights during training. Bottom: evolution of model performance during training. Synaptic weight denotes the strength of granule cell → Purkinje neuron synapses. Ipsilateral synapses connect stimulus-activated granule cells (left or right) to Purkinje neurons encoding the same side, whereas cross synapses connect stimulus-activated granule cells to Purkinje neurons encoding the opposite side. The weakening of cross synapses accounts for the improved model performance.

SI References

1. J. Touboul, R. Brette, Dynamics and bifurcations of the adaptive exponential integrate-and-fire model. *Biol. Cybern.* **99**, 319–334 (2008).
